# Anisotropic Masked Mycobacterium Potentiates Amplified Antitumor Trained Immunity via Spleen Targeting and Myelopoiesis Conversion

**DOI:** 10.1002/adma.74513

**Published:** 2026-08-05

**Authors:** Jin‐Ho Choi, Sang Nam Lee, Jang Hun Heo, Soyoung Park, Sei Hyun Park, Yong Taik Lim

**Affiliations:** ^1^ SKKU Advanced Institute of Nanotechnology (SAINT) Department of Nano Science and Technology Department of Nano Engineering School of Chemical Engineering and Biomedical Institute For Convergence at SKKU Sungkyunkwan University Suwon Republic of Korea

**Keywords:** preoperative treatment, splenic myelopoiesis, TLR agonist, trained immunity, tumor recurrence

## Abstract

Cancer progression involves systemic immune suppression and tumor recurrence facilitated by increased splenic protumoral myelopoiesis. To address this clinical challenge, we developed an engineered Trojan Mycobacterium as a trained immunity (TI)‐mediated splenic myelopoiesis converter (t‐SMC). This design provides an intravenously injectable anisotropic TI inducer with an aspect ratio of 3.6 for preferential spleen targeting and cellular uptake via a Mycobacterium “peeling‐off and masking‐up” strategy with human serum albumin incorporating an enzyme‑activatable Toll‑like receptor 7/8 agonist that boosts TI activation. t‐SMC induces antitumoral myelopoiesis with metabolic and epigenetic reprogramming in splenic macrophages. This shift toward antitumoral myelopoiesis markedly enriches the tumor microenvironment with inflammatory myeloid effectors in an E.G7‐OVA model, and the adoptive transfer of splenic myeloid cells confirmed the TI‐driven antitumor effect. Furthermore, comparative analysis revealed that the bacterial scaffold (alb‐Mycobac) initiates myeloid reprogramming, but only the integrated TLR7/8 agonist (t‐SMC) crosses the therapeutic threshold for tumor regression. In the MC38 surgical recurrence model, preoperative t‐SMC combined with standard adjuvant αPD‐L1 plus oxaliplatin achieved complete inhibition of recurrence in 50% of treated mice (3 of 6) with 62.5% long‐term survival, establishing TI priming as a translation‐ready preoperative adjuvant that converts immune‐checkpoint non‐responders into responders.

## Graphical abstract

An engineered Trojan Mycobacterium, developed via a “peeling‐off and masking‐up” strategy, functions as a powerful splenic myelopoiesis converter. This spleen‐targeted nanomedicine induces trained immunity to reprogram immunosuppressive myeloid reservoirs into antitumoral effectors. Preoperative administration effectively thwarts postsurgical tumor recurrence and sensitizes refractory landscapes to checkpoint blockades.

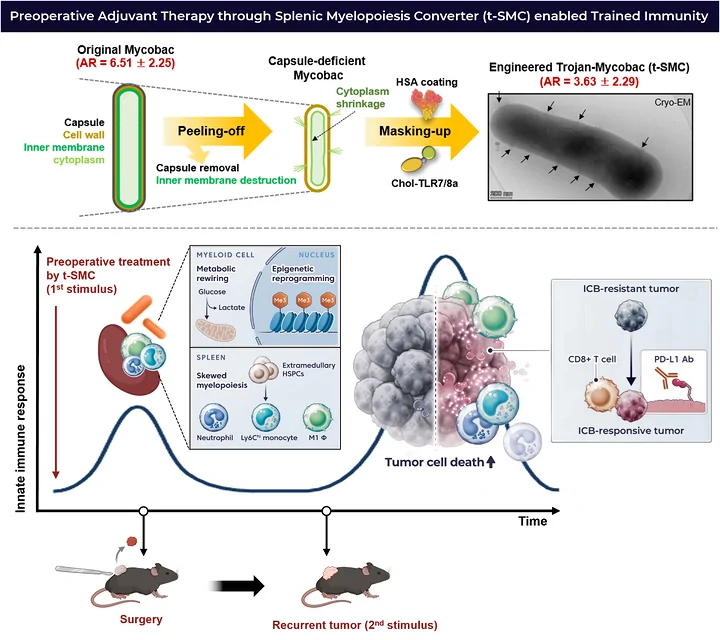

## 1 Introduction

Protumoral splenic myelopoiesis and subsequent systemic immunosuppression drive postsurgical recurrence, significantly reducing 5‐year survival rates [1, 2]. Thus, the induction of antitumoral myelopoiesis in the spleen by harnessing trained immunity (TI), which provides non‐specific, enhanced innate immune responses to subsequent challenges, is likely to be crucial for the success of cancer immunotherapy [3, 4, 5, 6, 7, 8].

While many TI‐inducing strategies focus on bone marrow [9, 10], targeting the spleen offers distinct advantages for systemic immune modulation. The spleen serves as a critical “myeloid hub” that undergoes significant remodeling during tumor progression, characterized by the accumulation of a splenic monocyte reservoir and the onset of extramedullary hematopoiesis (EMH) [11]. In the context of malignancy, this splenic environment often acts as a pro‐tumoral nexus, where EMH typically drives the generation and storage of immunosuppressive myeloid cells, which subsequently infiltrate the tumor microenvironment (TME) to facilitate immune evasion [12, 13, 14].

However, this splenic reservoir also presents a unique therapeutic opportunity. Emerging evidence suggests that TI inducers can effectively recruit and reprogram pro‐tumoral hematopoietic stem and progenitor cells (HSPCs) within the spleen [15, 16, 17]. By shifting these progenitors toward a pro‐inflammatory myeloid phenotype, TI induction enhances their intrinsic antitumor capacity. Once reprogrammed, these splenic myeloid cells migrate to the TME, transforming the local landscape from an immunosuppressive state to one that is actively antitumoral. Furthermore, this splenic reprogramming bridges innate and adaptive sectors by promoting T cell recruitment and activation, orchestrating a robust systemic antitumor response. Consequently, spleen‐targeted TI inducers offer a scalable strategy to potentiate standard immunotherapies through multifaceted immune coordination.

Here, we propose an engineered Trojan horse‐like Mycobacterium that functions as a TI‐mediated splenic myelopoiesis converter (t‐SMC). In this study, we utilized Bacillus Calmette‐Guérin (BCG), a known inducer of TI, as the core training agent. Autoclaved BCG (hereafter referred to as ‘Original Mycobac’) was engineered to reduce residual toxicity by applying a human serum albumin (HSA) surface coating to yield a formulation suitable for intravenous (IV) administration while concurrently integrating a cholesterol‐conjugated and γ‐interferon‐inducible lysosomal thiol reductase (GILT) enzyme‑activatable Toll‑like receptor 7/8 agonist (resiquimod) to boost TI (Chol‐TLR7/8a) (Figure 1a; Figures S1–S3). Previous studies have demonstrated that Chol‐TLR7/8a reduces systemic toxicity through a masking mechanism and maximizes immunostimulatory activity by kinetically modulating the activation profiles of innate immune cells [18, 19]. After the peeling‐off and masking‐up engineering process, t‐SMCs form a large and anisotropic shape with enhanced colloidal stability, enabling stable aqueous dispersion. Also, the aspect ratio (AR) of 3.6 induced by cytoplasmic shrinkage is preferential for spleen targeting and cellular uptake. Eventually, IV‐administered t‐SMCs access and engage splenic myeloid niches, augment local training signals through inflammatory cytokine secretion (TNF‐α and IL‐6), and thereby redirect myelopoiesis towards antitumoral phenotypes (Figure 1b). We further report that the t‐SMC facilitates TI through metabolic and epigenetic reprogramming. This enables the recognition of recurrent tumors as secondary stimulation, thereby enhancing the antitumor efficacy of splenic myeloid cells (Figure 1c). Increased infiltration of trained splenic myeloid cells within the TME converts the immunosuppressive microenvironment into an inflammatory antitumor environment and effectively suppresses tumor recurrence. Furthermore, t‐SMC enhanced immune checkpoint blockade (ICB) responsiveness of TME, resulting in more effective suppression of cancer recurrence. Notably, t‐SMCs administered as a preoperative adjuvant with standard chemotherapy and ICB effectively prevented tumor relapse. Unlike previous studies that applied TI as a postoperative treatment or rechallenge model [20, 21], this work establishes a novel approach to overcoming ICB resistance and tumor regrowth by introducing a TI‐based preoperative treatment concept to a recurrence model.

**FIGURE 1:**
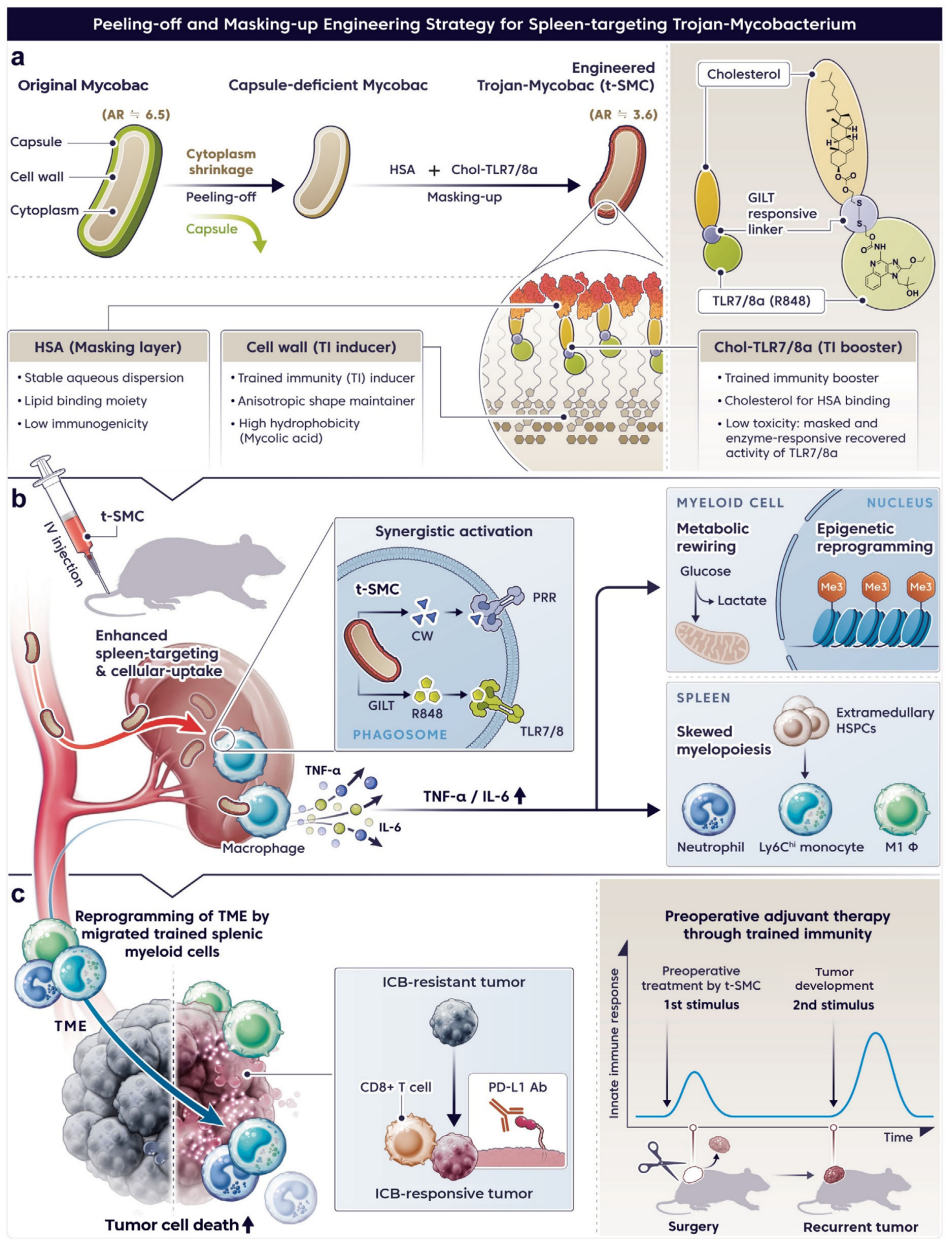
Overview of the synthesis, delivery, and antitumor mechanisms of the trained immunity‐mediated splenic myelopoiesis converter (t‐SMC). (a) Schematic representation of Mycobacterium engineering process using a peeling‐off and masking‐up strategy. (b) Effects of t‐SMC in the spleen. Cell wall, CW; H3K4me3, Me3. (c) Enhancement of antitumor effects via infiltration of trained splenic myeloid cells into TME and use of t‐SMC as a preoperative adjuvant in the surgical model.

## 2 Results

### 2.1 Engineering BCG by a Peeling‐Off and Masking‐Up Strategy

While BCG IV injection induces potent systemic TI [22, 23], the risk of multi‐organ dissemination and sepsis restricts its clinical use to local administration [24]. Furthermore, the use of live Mycobacteria in medications is challenging due to aggregation issues, which cause inconsistent dosing, injection clogging, safety risks like embolism, and variable efficacy [25, 26, 27]. The “peeling‐off and masking‐up” strategy has broader applicability to other bacterial species such as Mycobacteria with polysaccharide capsular layers and Gram‐negative species with LPS‐bearing outer envelopes. After peeling‐off, the hydrophobic bacterial cell wall surface was exposed (Capsule‐deficient Mycobac) (Figure 2a,b). Then, HSA was employed as a surface‐masking agent for Capsule‐deficient Mycobac, markedly improving its colloidal stability in aqueous media. To further enhance TI, Chol‐TLR7/8a, which engages HSA through lipid‐binding pockets, was also incorporated into the formulation. As a result, anisotropic and large particles that remained readily dispersible in aqueous solution formed (Figure 2c). To investigate the spatial organization of t‐SMCs, we performed confocal laser scanning microscopy (CLSM) using fluorescently labeled components. Prior to t‐SMC synthesis, HSA was labeled with an Alexa Fluor 488‐conjugated anti‐HSA antibody, while Cy5‐conjugated cholesterol (Chol‐Cy5) was introduced instead of Chol‐TLR7/8a during synthesis. CLSM imaging revealed that the HSA signal was predominantly localized to the outer shell. These results demonstrate that HSA effectively coats the internal cargo, including Capsule‐deficient Mycobac and Chol‐TLR7/8a, within the t‐SMC structure (Figure 2d). Furthermore, using HSA‐bearing AuNP labels and cryo‐electron microscopy (cryo‐EM), the presence of HSA on the Mycobacterium surface was confirmed (Figure 2e). In cryo‐EM imaging, Original Mycobac was highly clumped due to the combined effects of its intrinsic nature and heat‐induced surface modifications [28]. In contrast, alb‐Mycobac and t‐SMC demonstrated superior dispersibility, maintaining a uniform distribution in water without a tendency to aggregate, as evidenced by their cryo‐EM images and polydispersity index (PDI) values (Figure 2f; Figure S4a–d). Capsule removal induced physical changes, including membrane loss, leading to cytoplasm shrinkage (Figure 2g,h) [29]. Consequently, the cell dimensions decreased, and the mean AR was reduced from 6.51 ± 2.25 to 3.63 ± 2.29, a range favorable for efficient cellular uptake [30, 31, 32]. Additionally, cryo‐EM analyses revealed no morphological differences between alb‐Mycobac and t‐SMC (Figure 2f).

**FIGURE 2:**
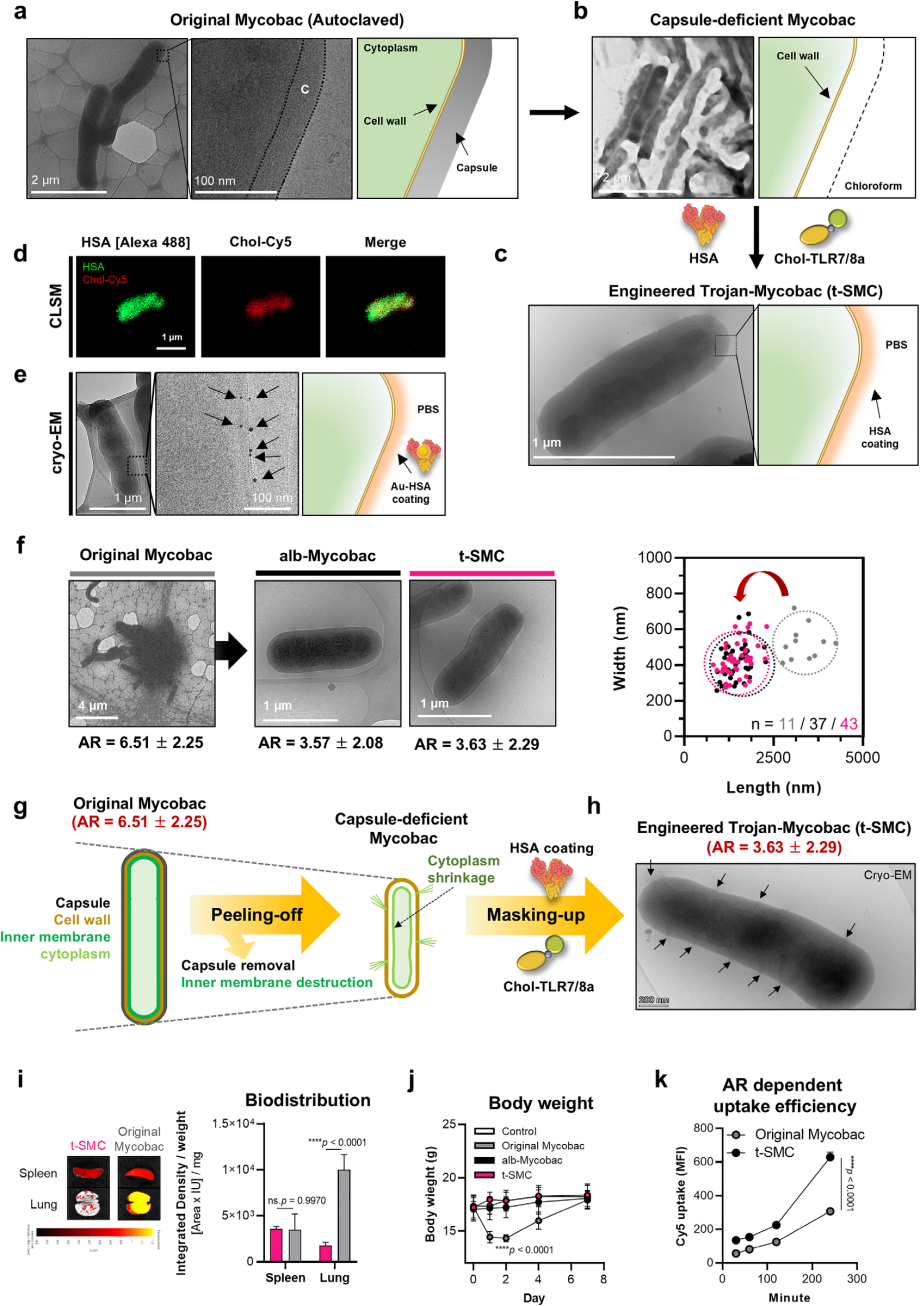
Synthesis of t‐SMC. (a) Cryo‐EM image of Original Mycobac. The region labeled ‘C’ represents the capsule. (b) HRTEM image of Capsule‐deficient Mycobac. (c) Cryo‐EM image of t‐SMC. d) Representative images of t‐SMC via CLSM. (e) Cryo‐EM image of t‐SMC using HSA‐bearing AuNP labels. The arrow indicates a AuNP. (f) Determination of the aspect ratio (AR) on the basis of the cryo‐EM images of Original Mycobac, alb‐Mycobac, and t‐SMC and particle distribution, with dots representing the measured length and width. (g) Schematic illustration of morphological changes after the Mycobac engineering process. h) Cytoplasmic leakage of synthesized t‐SMC after Mycobac engineering due to cytoplasm shrinkage, with black arrows indicating shrinkage spots. (i) Biodistribution analysis of t‐SMC and Original Mycobac 24 h after IV injection (*n* = 3 per group). (j) Changes in body weight after IV injection (*n* = 4 per group). (k) Comparison of cellular uptake efficiency of Original Mycobac and t‐SMC (*n* = 3 per group). All the data are presented as the mean ± s.d. Statistical significance was evaluated by two‐way ANOVA with Tukey's multiple‐comparison test in i, j, and k. *p*‐ values: ns, not significant; ****p < 0.0001.

To effectively induce TI, cells must internalize the inducer and subsequently secrete inflammatory cytokines such as TNF‐α or IL‐6 to modulate the surrounding immune environment. For the evaluation of t‐SMC‐mediated inflammatory cytokine secretion, RAW264.7 cells were treated with alb‐Mycobac, HSA+Chol‐TLR7/8a alone, or t‐SMC. After 24 h, the supernatant was collected to measure cytokine secretion (Figure S5a,b). The results demonstrated that cytokine secretion was significantly greater in the t‐SMC‐treated group than in the groups treated with the individual substances alone, indicating that t‐SMC more effectively induces inflammatory cytokine secretion.

In biodistribution analysis, t‑SMC preferentially accumulated in the spleen, whereas Original Mycobac was predominantly localized in the lungs as well as the spleen, indicating a non‑specific organ targeting pattern, while alb‐Mycobac and t‐SMC shared a similar biodistribution (Figure 2i; Figure S6a,b). Furthermore, Original Mycobac caused significant body weight loss post‐injection, while mice treated with alb‐Mycobac or t‐SMCs maintained stable weights, confirming the improved biocompatibility of our engineering strategy (Figure 2j). Additionally, IV injection of alb‐Mycobac and t‐SMC showed negligible hepatotoxicity, with serum AST and ALT levels rapidly returning to baseline (Figure S7a). These results suggest that systemic TI was induced without sustained systemic inflammation, as the absence of a secondary stimulus likely prevented an unintended recall response. Histopathological examination of tissues using H&E staining revealed no evidence of tissue damage (Figure S7b). In biological systems, cellular uptake is maximized for anisotropic particles with an AR of 3‐4 than spherical ones (AR = 1), whereas efficiency decreases for highly elongated (AR>4) shapes [31, 32]. Consistent with this, Cy5‐labeled t‐SMCs exhibited more than 2‐fold greater cellular uptake in RAW264.7 cells than Original Mycobac (Figure 2k). Together, these findings suggest that our BCG engineering strategy successfully improved the biocompatibility, spleen‐specific delivery capability, and cellular uptake efficiency.

### 2.2 t‐SMC Drives HSPCs Reprogramming as a Splenic Myelopoiesis Converter

Biodistribution analysis after IV injection revealed that t‐SMC fluorescence was present in both the liver and spleen at 1 h, consistent with rapid first‐pass uptake by the mononuclear phagocyte system. However, kinetic analysis over 72 h showed that while the hepatic signal declined sharply due to rapid clearance, the splenic signal remained essentially stable (Figure 3a–c). This prolonged persistence in the spleen is attributed to its anisotropic structure (AR = 3.6), a geometry known to enhance splenic retention [30].

**FIGURE 3:**
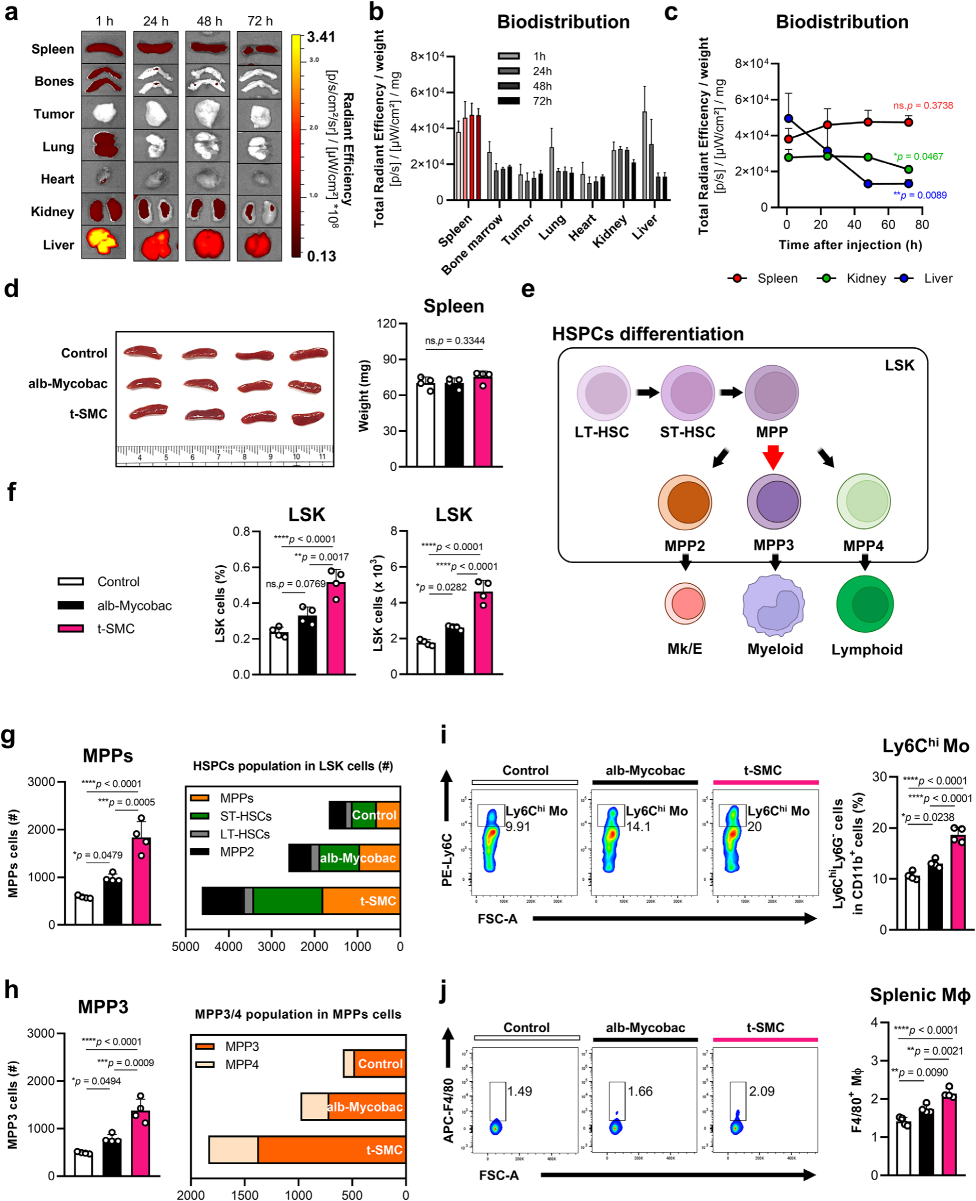
Splenic HSPCs reprogramming effect of t‐SMC. (a) Biodistribution analysis of t‐SMC for 72 h after IV injection using an IVIS (*n* = 3 per group). (b) Fluorescence intensity values of each organ at each time point after IV injection. (c) Fluorescence intensity profiles of the spleen, liver, and kidney following IV injection. Statistical significance was analyzed between the 1 h and 72 h time points within each organ. d) Spleen images and weights at 7 days after IV injection (*n* = 4 per group). e) HSPCs differentiation. Megakaryocyte, Mk; Erythroid, E. (f–j) Analysis of the spleens at 7 days after IV injection of the indicated materials into C57BL/6 mice (*n* = 4 per group): (f) population (left) and numbers (right) of lineage^−^Sca1^+^c‐Kit^+^ (LSK) cells; (g) numbers of multipotent progenitors (MPPs; LSK^+^CD150^−^CD48^+^); (h) numbers of MPP3 (LSK^+^CD150^−^CD48^+^Flt3^−^); (i) population of CD11b^+^Ly6G^−^Ly6C^high^ monocytes; (j) population of CD11b^+^Ly6G^−^Ly6C^−^F4/80^+^ macrophages. All the data are presented as the mean ± s.d. Statistical significance was evaluated by one‐way ANOVA with Tukey's multiple‐comparison test in c, d, and f–j. *p* values: ns, not significant; *p < 0.05; **p < 0.01; ***p < 0.001; ****p < 0.0001.

To evaluate whether t‐SMC administration reprograms splenic HSPCs into TI‐mediated myelopoiesis, we analyzed splenic tissues 7 days after IV injection. While spleen weights did not significantly differ between groups (Figure 3d), HSPCs characterization revealed significant changes in various subpopulations (Figure 3e). Lineage^−^Sca1^+^c‐Kit^+^ (LSK) cell populations and their numbers in both the alb‐Mycobac and t‐SMC groups were increased (Figure 3f), and greater numbers of multipotent progenitor cells (MPPs; LSK^+^CD150^−^CD48^+^) were observed in both treatment groups than in the control group (Figure 3g). Compared to alb‐Mycobac, the t‐SMC group exhibited a more pronounced expansion of MPPs. While both treatments increased MPP3 (myeloid‐biased) and MPP4 (lymphoid‐biased) populations, t‐SMCs induced a significantly greater MPP3 increase, indicating a stronger myeloid bias (Figure 3h). Consequently, both groups showed elevated inflammatory Ly6C^hi^ monocytes and splenic macrophages expressing high CD80 and CD86 co‐stimulatory receptors (M1‑like), with the most substantial increases observed in the t‐SMC group (Figure 3i,j; Figure S8a,b). These results indicate that t‐SMC treatment leads to enhanced inflammatory myelopoiesis as a splenic myelopoiesis converter (SMC).

### 2.3 t‐SMC Drives TI in Splenic Myeloid Cells

The key indicators of TI, such as metabolic and epigenetic reprogramming and enhanced responsiveness to secondary heterologous stimuli, were assessed by sorting splenic macrophages 7 days after IV injection (Figure 4a). Metabolic rewiring is a hallmark of TI and typically involves increased glycolysis [33]. To evaluate these processes, we measured lactate production by splenic macrophages from the alb‐Mycobac and t‐SMC groups and found that compared with the control group, both treatments resulted in higher lactate levels, indicating enhanced glycolytic activity characteristic of TI (Figure 4b).

**FIGURE 4:**
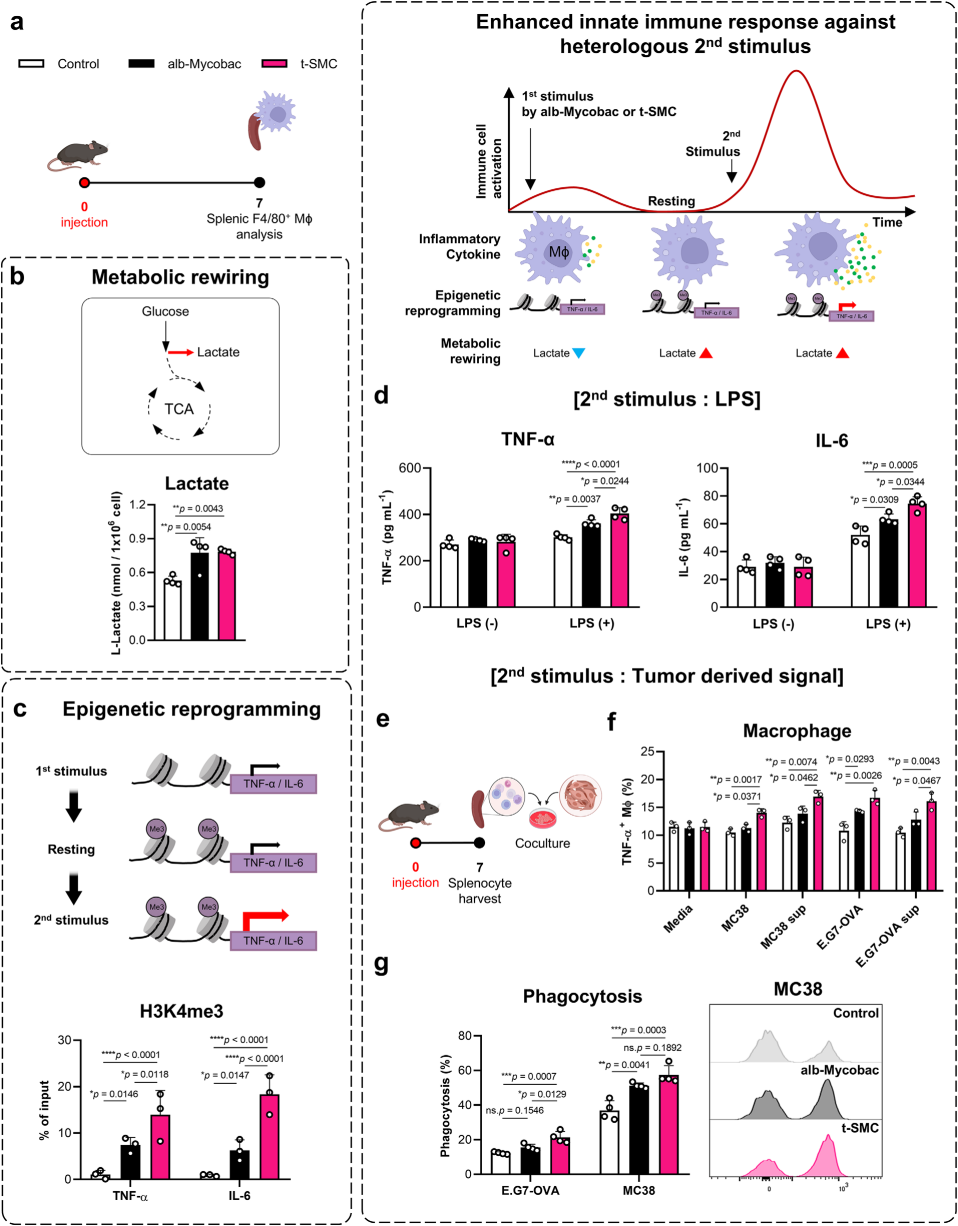
t‐SMC induced splenic myeloid cell trained immunity. a) F4/80^+^ macrophages in the spleen were sorted and analyzed 7 days after IV injection. b) Assessment of cellular metabolism by a lactate assay (n=4 per group). c) Evaluation of H3K4me3 methylation at the *Tnf* and *Il6* promoters by ChIP‒qPCR (n=3 per group). d) Measurement of cytokine (TNF‐α and IL‐6) secretion after LPS restimulation (n=4 per group). e) 7 days after IV injection, splenocytes were cocultured with tumor cells or tumor cell supernatant. f) Analysis of TNF‐α^+^ macrophages following restimulation by coculture with tumor cells (E.G7‐OVA or MC38) and the tumor cell supernatant (*n* = 3 per group). (g) Evaluation of the tumor cell phagocytosis capacity of splenic macrophages (*n* = 4 per group). All the data are presented as the mean ± s.d. Statistical significance was evaluated by one‐way ANOVA with Tukey's multiple‐comparison test in b–d and f–g. *p* values: ns, not significant; *p < 0.05; **p < 0.01; ***p < 0.001; ****p < 0.0001.

To assess epigenetic reprogramming, we performed ChIP‐qPCR for H3K4me3, a marker of active transcription and TI at the *Tnf* and *Il6* promoters (Figure 4c) [34]. While both alb‐Mycobac and t‐SMC groups showed increased H3K4me3 enrichment, the t‐SMC group exhibited significantly higher levels than the alb‐Mycobac group.

To assess the functional impacts of these metabolic and epigenetic changes, we treated trained macrophages with LPS as a second heterologous stimulus (Figure 4d) [3, 35, 36, 37, 38, 39]. Compared with the control group, both the alb‐Mycobac group and the t‐SMC group secreted higher levels of inflammatory cytokines (TNF‐α and IL‐6), with the t‐SMC group showing the most pronounced increases. These data suggest a heightened immune response to heterologous stimulation. We also explored whether trained splenic myeloid cells respond to tumor‐derived signals. Trained splenocytes were cocultured with MC38 and E.G7‐OVA tumor cells, which resulted in increased populations of TNF‐α^+^ macrophage, monocyte, and myeloid cells (Figures 4e,f; Figure S9a,b). Similar results were observed when cells were cocultured with tumor cell supernatants, indicating that TI can be triggered by both tumor cells and their secreted factors. Additionally, trained splenic macrophages exhibited increased phagocytosis against tumor cells, suggesting a direct role in antitumor activity (Figure 4g).

In this study, Capsule‐deficient Mycobac was utilized not only as a potent inducer of trained immunity but also as a structural framework to maintain an anisotropic configuration for effective splenic delivery. In our ex vivo splenocyte restimulation assay, mice treated with Chol‐TLR7/8a liposomes did not exhibit an increase in TNF‐α^+^ myeloid cells upon LPS restimulation, whereas both the alb‐Mycobac and t‐SMC groups showed significant elevations (Figure S10a). To further elucidate these effects, we performed an in vitro assessment using bone marrow‐derived macrophages (BMDMs) (Figure S10b). We compared the secretion of TNF‐α across different treatment groups following a heterologous secondary stimulus to evaluate their respective capacities for inducing innate immune memory. Our experimental results revealed that the t‐SMC group exhibited the highest levels of cytokine production, significantly outperforming the other groups, followed by the alb‐Mycobac group. Notably, the group treated with Chol‐TLR7/8a alone exhibited TNF‐α secretion comparable to control levels. These findings suggest that the structural architecture of Capsule‐deficient Mycobac is not only crucial for splenic delivery, but also that Chol‐TLR7/8a independently is insufficient to elicit a robust trained immune response. Instead, when combined with alb‐Mycobac (a canonical trained immunity inducer), Chol‐TLR7/8a serves to synergistically potentiate the trained immune response. This interaction, effectively integrated within the t‐SMC framework, ultimately culminates in a substantially more potent trained immunity effect than that achieved by any single component alone.

### 2.4 t‐SMC Induces an Enhanced Antitumor Effect via TI

To investigate whether the TI induced by t‐SMC results in antitumor effects, we challenged a subcutaneous (SC) E.G7‐OVA tumor model. The mice received an IV injection of PBS, alb‐Mycobac, and t‐SMC, and the tumor was inoculated 7 days later (Figure 5a). The t‐SMC group showed significant tumor growth inhibition compared with the control group (Figure 5b,c), with the t‐SMC group showing a marked reduction in tumor weight (Figure 5d).

**FIGURE 5:**
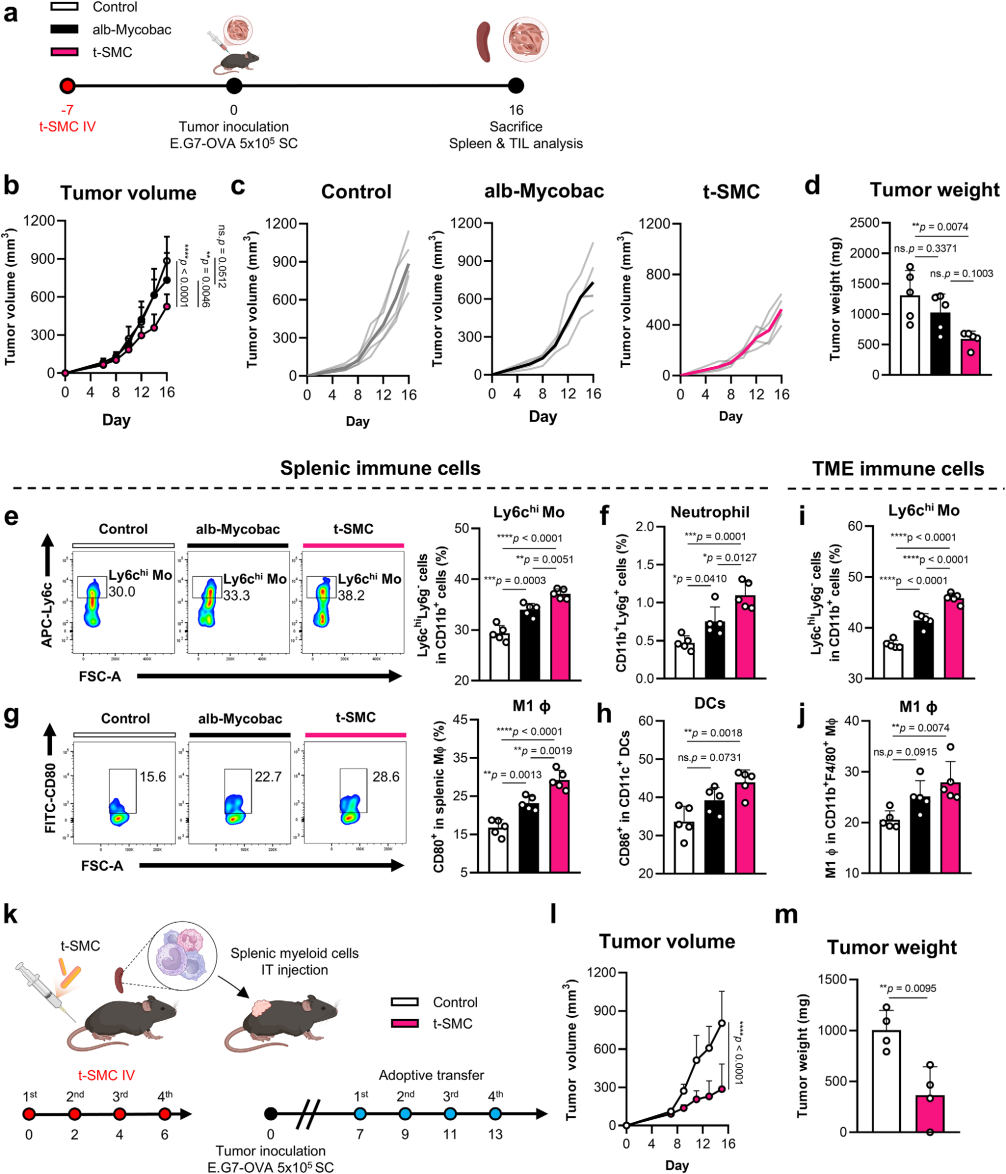
Trained immunity‐mediated tumor suppression induced by t‐SMC. (a–d) Experimental schedule involving alb‐Mycobac and t‐SMC via IV administration followed by subcutaneous (SC) injection of E.G7‐OVA tumor cells 7 days later (*n* = 5 per group): (a) treatment timeline as shown, (b) tumor volume of each group, (c) individual tumor growth curves over time, and (d) tumor weight. (e–h) Analysis of splenic innate immune cell populations: (e) Ly6C^hi^ monocytes, (f) neutrophils, (g) M1 macrophages, and (h) dendritic cells (DCs). (i, j) Analysis of the following innate immune cell populations in the tumor microenvironment (TME): (i) Ly6C^hi^ monocytes and (j) M1 macrophages. (k) Splenic myeloid cell adoptive‐transfer schedule. (l) Tumor volume of each group (*n* = 4 per group). (m) Tumor weights. All the data are presented as the mean ± s.d. Statistical significance was evaluated by two‐way ANOVA with Tukey's multiple‐comparison test in b and l, one‐way ANOVA with Tukey's multiple‐comparison test in d, e‐j, and unpaired two‐tailed t test in m. *p* values: ns, not significant; *p < 0.05; **p < 0.01; ***p < 0.001; ****p < 0.0001.

Analysis of splenic immune cell populations revealed significantly increased inflammatory Ly6C^hi^ monocytes in the t‐SMC group (Figure 5e). Additionally, neutrophils, M1 macrophages, and CD86^+^ dendritic cells (DCs) populations were elevated in the t‐SMC group (Figure 5f–h). Although alb‐Mycobac alone was insufficient to drastically reduce tumor volume, splenic immune population analysis revealed that it played a substantial role in systemic reprogramming. Compared to the control, alb‐Mycobac significantly increased the population of Ly6C^hi^ monocytes, neutrophils, and M1‐like macrophages within the spleen. Examination of immune cell populations in the TME also revealed pronounced increases in Ly6C^hi^ monocytes and neutrophils in alb‐Mycobac group and t‐SMC group (Figure S11a). Notably, the t‐SMC group also exhibited a significant rise in M1 macrophages within the TME, while no significant change in M2 macrophages was observed across the groups (Figure 5i,j; Figure S11b). These findings suggest that while the alb‐Mycobac effectively initiates the reprogramming of splenic myeloid cells into an inflammatory phenotype, the addition of the Chol‐TLR7/8a in t‐SMC is essential to amplify this trained immunity to a threshold that translates into definitive tumor regression.

To determine whether the observed antitumor effects were predominantly mediated by trained immune cells, splenic myeloid cells were sorted from trained mice and adoptively transferred into tumor‐bearing, untrained recipients (Figure 5k). As a result, trained splenic myeloid cell recipients exhibited markedly suppressed tumor growth (Figure 5l,m).

Underlying the antitumor effects of myeloid cells trained by t‑SMC, the transcriptomic alterations were investigated by sorting trained splenic myeloid cells. Compared with the control group, the trained myeloid cells exhibited a globally altered gene expression profile. Volcano plot analysis revealed upregulation of 217 genes and downregulation of 187 genes (Figure 6a). Consistently, hierarchical clustering demonstrated high intra‑group similarity and a distinct inter‑group separation in gene expression patterns (Figure 6b).

**FIGURE 6:**
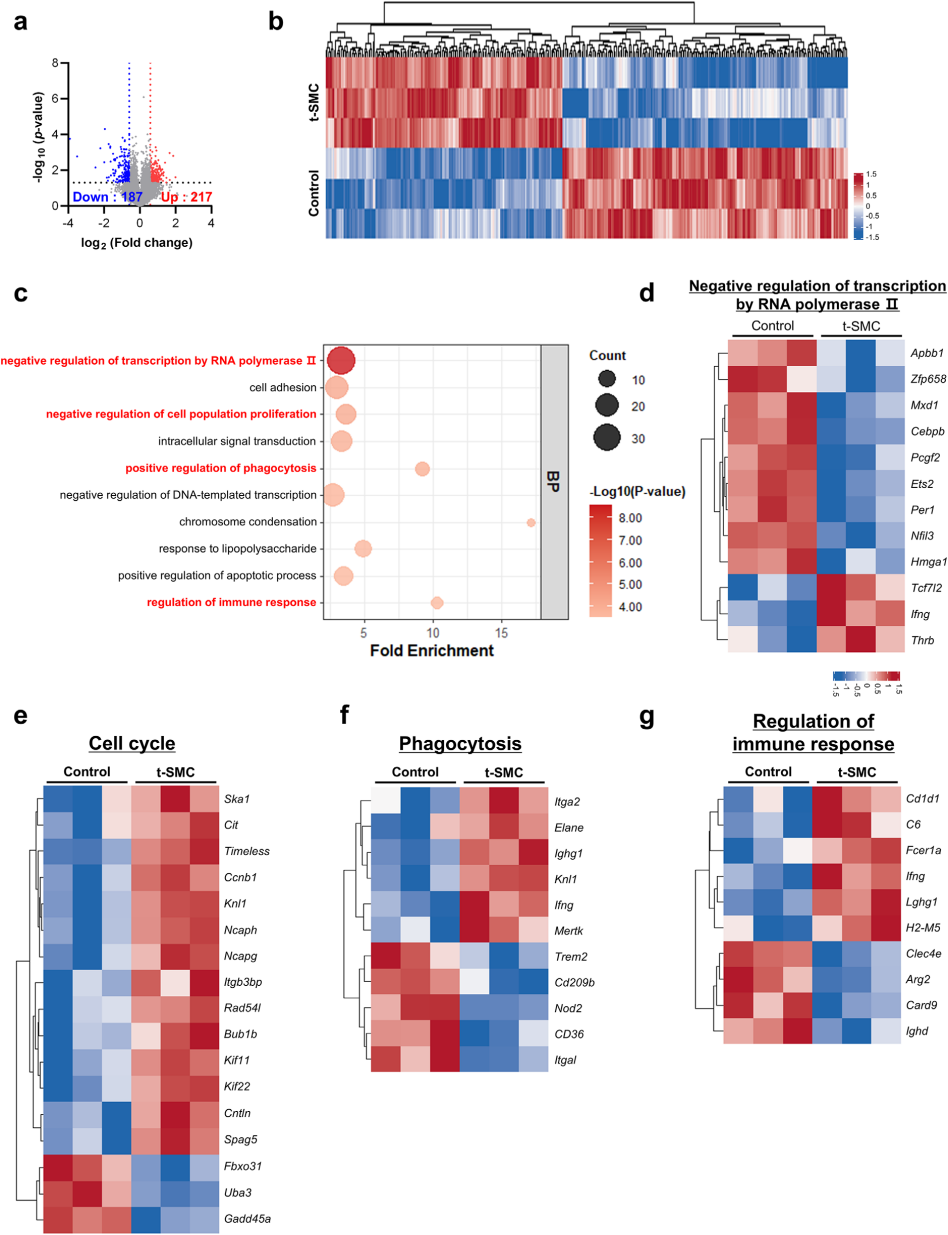
Transcriptional analysis of trained immunity effects on splenic myeloid cells induced by t‐SMC. (a) Volcano plot displaying significantly up‐regulated and down‐regulated between Control and t‐SMC treated splenic myeloid cells. The vertical lines denote a ± fold change of  1.5, and the horizontal lines denote a *p* value of 0.05. The x‐axis represents the log_2_ of the fold change, and the y‐axis represents the—log_10_ of the *p* value. (b) Heatmap clustering showing expression levels for a subset of genes that were differentially expressed in splenic myeloid cells between Control and t‐SMC treated mice. (c) Gene Ontology terms most strongly enriched among differentially expressed genes regulated by t‐SMC in splenic myeloid cells. (d–g) Heatmap of genes related to (d) negative regulation of transcription by RNA polymerase II, (e) cell cycle, (f) phagocytosis, and (g) regulation of immune response.

Gene Ontology analysis revealed genes associated with the negative regulation of transcription by RNA polymerase II were most significantly enriched, closely followed by cell proliferation, phagocytosis, and immune response (Figure 6c). Heatmap clustering revealed significant downregulation of RNA polymerase II inhibitory factors, indicating reduced transcriptional interference and enhanced transcriptional efficiency across affected pathways (Figure 6d). In alignment with this transcriptional shift, a notable decrease was observed in genes responsible for the negative regulation of cell proliferation (Figure S12) and significant upregulation of markers associated with the cell cycle (Figure 6e), collectively indicating that the cell population was undergoing active proliferation. Additionally, the increased expression of *Mertk* and *Ifng* suggests a dual functional remodeling, encompassing both enhanced efferocytic clearance (via *Mertk*) and pro‐inflammatory activation (via *Ifng* signaling) (Figure 6f). Furthermore, the clustering heatmap of regulation of immune responses exhibited significant decrease in *Arg2* expression, which is associated with anti‐inflammatory macrophage programming and arginine metabolism–associated immune suppression within the TME (Figure 6g), suggesting reduced tumor‐mediated immune evasion [40, 41]. This was accompanied by elevated *H2‐M5* and *Cd1d1* levels, critical for T and NKT cell activation, alongside upregulation of inflammatory mediators (*Ifng*, *C6*), indicating a shift toward an antitumoral inflammatory myeloid phenotype.

Additionally, analysis of adaptive immune populations revealed that the t‐SMC group exhibited significantly higher frequencies of antigen‐specific CD8^+^ T cells (Ag CD8^+^ T cells) in both spleen and TME compared to the control group, whereas T_reg_s remained stable, avoiding the typical elevation observed as a side effect of conventional BCG therapy. (Figure S13a–f). Furthermore, type 1 helper T (T_h1_) cells were elevated in the spleen in both the alb‐Mycobac and t‐SMC groups (Figure S13g), suggesting that trained myeloid cells may have contributed to the activation of adaptive immune system in the spleen. To assess adaptive contributions, we performed CD8^+^ T cell depletion (Figure S13h). While depletion reduced the magnitude of tumor inhibition compared to t‐SMC alone, growth remained partially suppressed relative to controls (Figure S13i).

Collectively, these results establish that t‐SMC‐induced tumor suppression is an innate‐driven process that subsequently orchestrates a robust adaptive immune response. While the initial reprogramming occurs in the splenic myeloid reservoir, as evidenced by the causative antitumor effect of adoptively transferred trained CD11b^+^ cells, this innate training acts as a critical functional bridge to the adaptive arm. The observed recruitment of Ag CD8^+^ T cells and the partial reduction in efficacy following CD8^+^ T cell depletion suggest that trained myeloid cells do not act in isolation. Instead, they remodel the immunosuppressive TME and enhance the priming of T cells, likely through improved antigen presentation and pro‐inflammatory cytokine secretion. Notably, the fact that tumor growth was still significantly restricted even in the absence of CD8^+^ T cells reinforces that innate effector function remains the primary and indispensable driver of t‐SMC therapy, with adaptive immunity serving to amplify and sustain the initial antitumor strike.

### 2.5 Preoperative Adjuvant t‐SMC Treatment Suppresses Tumor Recurrence in a Colon Cancer Surgical Resection Model

Despite its curative role, surgical resection triggers an immunosuppressive wound‐healing milieu that promotes tumor proliferation and local recurrence [42, 43]. Although ICB has been proposed as a surgical adjuvant, the low response rates of ICB limit its clinical success [44, 45]. We demonstrated that tumor cells act as secondary stimuli, triggering robust immune activation (Figure 4e–g; Figure S9a,b). To evaluate the clinical potential of t‐SMC‐mediated TI in preventing tumor recurrence, we preoperatively administered t‐SMCs to MC38 tumor‐bearing mice (Figure 7a). After resection, tumor recurrence in the t‐SMC group was significantly suppressed compared with the control group, as evidenced by slower tumor regrowth and lower tumor weights (Figure 7b–e).

**FIGURE 7:**
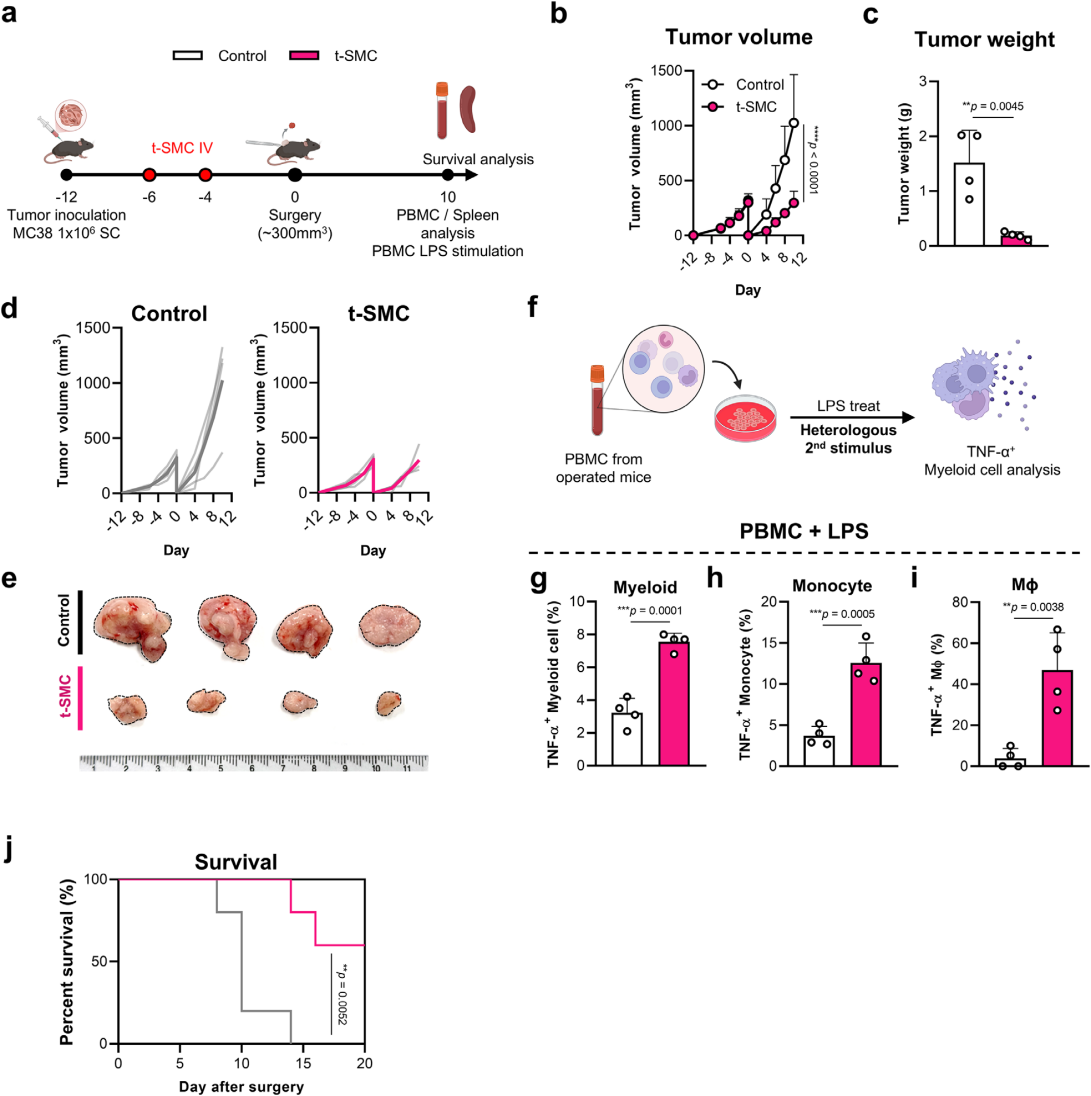
Preoperative adjuvant effect of t‐SMC in colon cancer surgical resection model. (a–e) Evaluation of t‐SMC‐mediated inhibition of tumor recurrence in the MC38 surgical model (*n* = 4 per group): (a) treatment schedule, (b) whole tumor volume of each group, (c) tumor weight of each group, (d) individual tumor growth curves over time, and (e) representative images of recurrent tumors. (f–i) Assessment of TNF‐α^+^ myeloid cell populations amongst PBMCs, which were harvested from mice 10 days after surgery, after LPS restimulation: (f) experimental scheme, (g) myeloid cells, (h) monocytes, and (i) macrophages. (j) Survival analysis of an independent cohort of the surgery model (*n* = 5 per group). All data are presented as the mean ± s.d. Statistical significance was evaluated by two‐way ANOVA with Tukey's multiple‐comparison test in b. Unpaired two‐tailed t tests were used in c and g–i. Survival *p* values were calculated by the log‐rank (Mantel–Cox) test in j. *p* values: **p < 0.01; ***p < 0.001; ****p < 0.0001.

Given the minimal tumor accumulation of t‐SMCs (Figure 3a), their anti‐recurrence effect likely stems from spleen‐derived trained cells. We confirmed this TI‐mediated response from operated mice by stimulating isolated peripheral blood mononuclear cells (PBMCs) with LPS to assess heterologous activation (Figure 7f). As a result, PBMCs from the t‐SMC group showed higher frequencies of TNF‐α^+^ myeloid cells, monocytes, and macrophages than the non‐trained group (Figure 7g–i). This implies that t‑SMC‐induced trained splenic myeloid cells can travel through the circulation and directly contribute to suppressing tumor recurrence. Additionally, the t‐SMC group demonstrated increased frequencies of Ag CD8^+^ T cell responses (IFN‐γ^+^ and TNF‐α^+^) in splenocytes, indicating activation of the systemic adaptive immune system (Figure S14a,b). Furthermore, analysis of effector memory CD8^+^ T cells (*T*_em_) and central memory CD8^+^ T cells (*T*_cm_) in the spleen revealed effective formation of immunological memory in the t‐SMC group (Figure S14c). Survival analysis of t‐SMC group also revealed an improved survival rate compared with the control group (Figure 7j). Collectively, these findings support that preoperative t‐SMC adjuvant treatment prevents tumor recurrence and improves survival in surgical models.

### 2.6 Combining ICB and Chemotherapy With Preoperative Adjuvant t‐SMC

We found that recurrent tumor (MC38) in the t‐SMC group exhibited higher PD‐L1 levels than those in the control group (Figure 8a). We therefore combined αPD‐L1 antibody treatment with t‐SMC to more effectively suppress tumor recurrence (Figure 8b). αPD‐L1 monotherapy had a minimal effect on tumor recurrence, whereas the combination of t‐SMC and the αPD‐L1 antibody led to significantly increased recurrence inhibition, including complete inhibition (1/4) (Figure 8c,d). Survival analysis further demonstrated that the ICB combination also markedly improved prognosis (Figure 8e).

**FIGURE 8:**
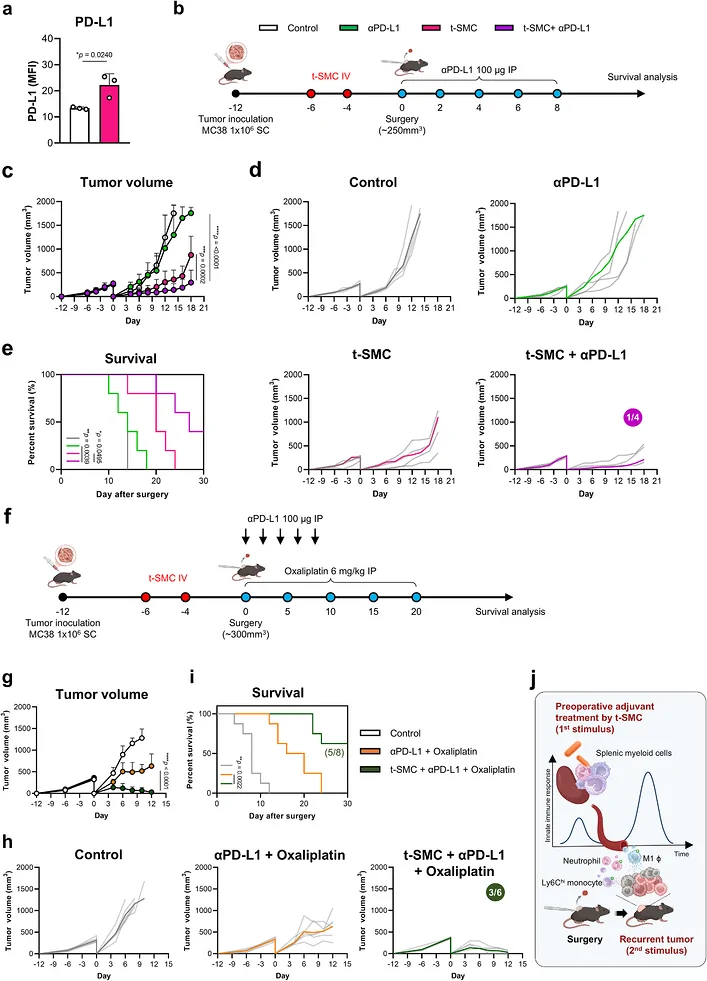
ICB and chemotherapy combination with preoperative adjuvant t‐SMC for tumor recurrence inhibition. (a) PD‐L1 expression in recurrent tumors 10 days after surgery (*n* = 3 per group). (b) αPD‐L1 combination therapy schedule (*n* = 4 per group). (c) Whole tumor volume of each group. (d) Individual tumor growth curves over time. e) Survival analysis of an independent cohort of the surgery model (*n* = 5 per group). (f) ICB and oxaliplatin combination therapy schedule (*n* = 6 per group). (g) Whole tumor volume of each group. (h) Individual tumor growth curves over time. (i) Survival analysis of an independent cohort of the combination therapy model (*n* = 8 per group). (j) Concept of preoperative adjuvant treatment with t‐SMC as a recurrent tumor suppressor. All data are presented as the mean ± s.d. Statistical significance was evaluated by unpaired two‐tailed t tests in a. Two‐way ANOVA with Tukey's multiple‐comparison test was used in c and g. Survival *p* values were calculated by the log‐rank (Mantel–Cox) test in e and i. *p* values: *p < 0.05; **p < 0.01; ***p < 0.001; ****p < 0.0001.

We next examined whether preoperative t‐SMC treatment suppresses tumor recurrence in a therapeutic setting mimicking clinical adjuvant chemotherapy, where postoperative oxaliplatin and ICB are administered to prevent recurrence (Figure 8f) [46, 47, 48, 49]. As a result, the combination therapy of αPD‑L1 antibody and oxaliplatin effectively suppressed tumor recurrence (Figure 8g). However, it did not completely eliminate recurrent lesions. By contrast, when preoperative t‑SMC treatment was combined, the recurrent tumor burden progressively decreased during the postoperative treatment phase, and complete recurrence inhibition was observed in a 50% subset of treated individuals (Figure 8h) with superior survival outcomes (Figure 8i).

Collectively, these findings suggest that t‐SMC, as a preoperative TI inducer, suppresses recurrence by enabling innate immune cells to recognize recurrent tumors as a secondary stimulus (Figure 8j). The infiltration of t‐SMC‐trained splenic myeloid cells successfully converts ‘cold' immunosuppressive landscapes into ’hot' inflamed environments, leading to an inflammatory milieu (TNF‐α, IL‐6, and IFN‐γ) that upregulates tumor PD‐L1 and sensitizes the TME to checkpoint blockade [9, 50, 51]. Thus, t‐SMCs act as a neoadjuvant primer that converts ICB non‐responders into responders and effectively prevents tumor recurrence.

## 3 Discussion

Cancer progression and recurrence are intricately linked to systemic immune suppression, a process largely driven by heightened splenic protumoral myelopoiesis. To address this, we developed a novel anisotropic bio‐hybrid design of a TI‐mediated splenic myelopoiesis converter (t‐SMC), an engineered Trojan horse‐like BCG masked with a HSA coating and equipped with a TLR 7/8a. t‐SMC has an optimized AR for preferential spleen targeting that reverses systemic immune suppression by converting splenic protumoral myelopoiesis into an antitumoral phenotype via TI. The ‘peeling‐off and masking‐up' strategy of BCG engineering produces colloidal stability and optimized AR (3.6) for spleen targeting. The specific anisotropic morphology facilitates efficient splenic accumulation and cellular uptake while minimizing off‐target deposition [30, 31, 32]. Unlike conventional bone‐marrow‐centric approaches, this spleen‐centered strategy leverages the organ's myeloid reservoir for systemic mobilization of trained antitumoral effectors. Eventually, IV administration of t‐SMC induces MPP3 population expansion (myelopoiesis) in HSPCs. Furthermore, enhanced glycolytic activity and H3K4me3 histone methylation in splenic macrophages, which are consistent with the hallmarks of TI were observed. Consequently, these trained myeloid cells exhibit increased cytokine secretion and superior tumor cell phagocytosis when restimulated by tumor‐derived signals, as well as antitumor effects by infiltrating into the TME.

Our comparative analysis reveals a hierarchical model of TI, wherein the alb‐Mycobac scaffold initiates systemic myeloid reprogramming but induces only intermediate metabolic and epigenetic changes insufficient for full tumor growth inhibition. In contrast, t‐SMC achieves robust tumor suppression by significantly amplifying lactate accumulation and H3K4me3 deposition at inflammatory cytokine promoters. These findings demonstrate that while the bacterial scaffold serves as the essential priming signal, the integrated TLR7/8 agonist provides the critical amplification required to cross the therapeutic threshold for definitive antitumor efficacy. This hierarchical synergy was validated in vitro in BMDMs, which decoupled the contributions of the agonist and the vehicle. While the Chol‐TLR7/8a + HSA‐treated BMDMs failed to elicit robust TI, the complete t‐SMC platform profoundly amplified TNF‐α output upon heterologous restimulation. This confirms that the TLR7/8 agonist alone is insufficient to drive innate memory. Instead, the bacterial matrix provides essential bacterial cell‐wall priming signals that synergize with the TLR7/8 agonist within the unified t‐SMC architecture, proving that this engineered bio‐hybrid system is the principal driver of the enhanced therapeutic response.

Strategically, preoperative t‐SMC offers a novel spleen‐centered paradigm to counteract surgery‐induced immunosuppression and recurrence. By establishing systemic immune memory before intervention, t‐SMC synergizes with oxaliplatin and ICB to overcome established resistance. While oxaliplatin primes the TME, t‐SMC ensures a sustained influx of antitumoral myeloid cells, achieving 50% complete regression in refractory models. To further clarify the clinical relevance and therapeutic superiority of our approach, we integrated an explicit benchmark comparison against the standard postoperative treatment window. In our study, the conventional doublet control group (αPD‐L1 + oxaliplatin) failed to achieve any complete responses in the MC38 recurrence model. This clinical failure serves as a critical baseline benchmark, reflecting how a profoundly immunosuppressive, surgery‐induced wound‐healing environment typically renders standard therapies ineffective. In stark contrast to this refractory baseline, the preoperative introduction of t‐SMC as a neoadjuvant primer successfully crossed the therapeutic threshold, driving 50% complete recurrence inhibition and significantly extending long‐term survival. This robust differentiation demonstrates that while conventional chemo‐immunotherapy alone is insufficient to dismantle the postsurgical myeloid barrier, the integration of t‐SMC serves as an indispensable driver that transforms a non‐responsive, ‘cold' tumor landscape into an inflamed, ICB‐sensitive ’hot' environment. These findings underscore the clinical potential of this chemo‐immunotherapeutic regimen. Furthermore, our ‘peeling‐off and masking‐up' strategy eliminates hydrophobic‐driven aggregation that persists despite stabilizers. This ensures colloidal stability and individual‐cell homogenization for predictable dosing, addressing major challenges in bacteria‐based translational medicine. This versatility across various strains enables broad applications beyond cancer therapy, including infectious diseases and inflammatory bowel disease [52, 53, 54, 55].

In summary, t‐SMC serves as a splenic myelopoiesis converter through its anisotropic design for preferential spleen targeting. By training splenic myeloid cells, t‐SMC shows clinical promise as a preoperative adjuvant that reduces recurrence and sensitizes tumors to ICB. Its ability to act synergistically with standard care makes it a versatile candidate for broad oncologic applications.

## 4 Methods

### 4.1 BCG engineering

Autoclaved *Mycobacterium bovis* BCG Korea 1168p (defined as Original Mycobac in this study) was obtained from Masan National Tuberculosis Hospital. Original Mycobac (1.6 × 10^8^ CFU) was suspended in 3 mL of PBS, and 42 mL of a 2 v% Triton X‐100 solution was combined with the bacterial suspension to reach a final volume of 45 mL. The mixture was subjected to ultrasonication in a preheated (90°C) sonic bath (Bransonic) for 2 h while being simultaneously boiled using a double boiler. After centrifugation at 10 000 × g for 20 min, the supernatant was removed, and the residual pellet was washed twice with 45 mL of acetone. The pellet was then resuspended in 3 mL of PBS and dispersed via tip sonication for 5 min. Then, 42 mL of 2% SDS solution was added to the dispersion, after which the total volume was adjusted to 45 mL. The mixture was reheated in a 90°C sonic bath, ultrasonicated for 2 h, and boiled again. After centrifugation under the same conditions, the supernatant was discarded, and the residue was subjected to two washes with acetone followed by a single wash with 45 mL of 10% v/v 2‐propanol. The final product was dispersed in 30 mL of ethanol, and the Capsule‐deficient Mycobac extract was purified as a powder through vacuum distillation to remove the ethanol. In our preparation, 300 µg of Capsule‐deficient Mycobac (alb‐Mycobac or t‐SMC) corresponded to approximately 1 × 10^7^ CFU of starting Original Mycobac, calculated based on the yield of approximately 4.8 mg of Capsule‐deficient Mycobac from the initial 1.6 × 10^8^ CFU.

### 4.2 GILT‐Dependent Chol‐TLR7/8a Analysis

To evaluate the GILT‐dependent cleavage of Chol‐TLR7/8a, nanoliposomes were prepared by encapsulating Chol‐TLR7/8a (equivalent to 50 µg of R848; reconstituted in 20 µL of PBS) based on reference [18]. For the cleavage assay, 2.5 µg of recombinant human GILT (IFI30, RayBiotech) was mixed with 50 µL of cysteine (1 µM) dissolved in PBS. This mixture was then added to the Chol‐TLR7/8a liposome suspension. The resulting solution was encapsulated in a dialysis membrane (MWCO 12 000, Sigma–Aldrich, D9277) and immersed in 10 mL of a 1 µM cysteine/PBS buffer. The samples were incubated in a shaking incubator at 37°C. At designated time points, the supernatant was harvested to quantify the free R848 released via GILT‐mediated cleavage. The concentration of the released R848 was analyzed using UV‒vis spectroscopy (UV‒1800) at its characteristic absorption wavelength.

### 4.3 Synthesis and Characterization of t‐SMC

Chol‐TLR7/8a was synthesized and characterized following Figures S1–S3. Capsule‐deficient Mycobac (8 mg mL^−^^1^) and Chol‐TLR7/8a (806 µM) were individually dispersed in chloroform and then combined at a 1:1 volumetric ratio. For alb‐Mycobac, pure chloroform without Chol‐TLR7/8a was used. HSA was dissolved in 40 mM L‐(+)‐lysine (TCI) in DW buffer at a concentration of 3%. The HSA buffer aqueous solution was gradually added to the organic chloroform mixture at a 1:4 (organic:aqueous) ratio. Emulsification via tip sonication (30% AMP, 1 min) preceded complete chloroform removal using a rotary evaporator (30 min, RT). After centrifugation (10 000 × g, 10 min, 4°C), the nanoparticles were washed twice with PBS (HyClone). The loading efficiency of Chol‐TLR7/8a was calculated to be approximately 70%, as determined by measuring the concentration of unencapsulated molecules in the supernatant via UV‒vis spectroscopy (UV‒1800). Particle characteristics were assessed using dynamic light scattering (DLS; ELS‐Z electrophoretic light scattering photometer) for hydrodynamic size and PDI, while morphology was examined via cryo‐TEM (Glacios 2, Sungkyunkwan University Cooperative Center for Research Facilities) and High Resolution Transmission (JEM ARM 200F, Sungkyunkwan University Cooperative Center for Research Facilities).

### 4.4 CLSM Imaging

To visualize the spatial distribution of the components within the t‐SMC, labeling was performed prior to synthesis. HSA was fluorescently tagged using an Alexa Fluor 488‐conjugated anti‐HSA antibody (IC1455G, R&D Systems) at a volume ratio of 1:20 (HSA antibody:3% HSA solution). The labeled HSA was then utilized to encapsulate Capsule‐deficient Mycobac and Chol‐Cy5 (used as a fluorescent surrogate for Chol‐TLR7/8a; R‐M‐C‐01, Ruixbio) to form the t‐SMC structure. The resulting complexes were characterized via CLSM (LSM 980 Airyscan 2, Sungkyunkwan University Cooperative Center for Research Facilities). Details of the antibody are provided in Table S1.

### 4.5 Animals, Cell Lines, and Antibodies

The animal experiments in this study received ethical approval following review by the Institutional Animal Care and Use Committee (IACUC) of Sungkyunkwan University School of Medicine (Protocol No: SKKUIACUC2024‐04‐18‐1). This committee operates in compliance with guidelines from the Institute of Laboratory Animal Resources (ILAR) and holds accreditation from the Association for Assessment and Accreditation of Laboratory Animal Care International (AAALAC International). 6‐week‐old female C57BL/6 mice were obtained from Orient Bio (Korea). The mice were maintained in individually ventilated cages with controlled environmental conditions (temperature of 21°C–26°C, humidity of 30%–70%, and a 12 h light/dark cycle). E.G7‐OVA murine T lymphoblasts (ATCC), MC38 murine colon adenocarcinoma cells (Sigma‒Aldrich), and RAW264.7 murine macrophages (ATCC) were cultured in either Dulbecco's modified Eagle's medium (DMEM; Gibco) or RPMI 1640 medium (Gibco), depending on the cell line requirements. The growth media contained 10% heat‐inactivated fetal bovine serum (FBS; Thermo Fisher), penicillin (50 IU mL^−1^), and streptomycin (50 µg mL^−1^; Thermo Fisher). Comprehensive details regarding the antibodies used, including fluorescence type, manufacturer, clone identifiers, and catalogue numbers, are given in Table S1.

### 4.6 Biocompatibility analysis

A 6‐week‐old female C57BL/6 mice were randomly assigned to receive intravenous injections of PBS (control), Original Mycobac (1 × 10^7^ CFU), alb‐Mycobac (300 µg of Capsule‐deficient Mycobac), or t‐SMC (300 µg of Capsule‐deficient Mycobac, equivalent to 21.17 nmol of R848). Body weight was monitored for 7 days following injection. For hepatotoxicity analysis, serum was harvested at 0, 6, 24, 72, 120, and 168 h postinjection, and aspartate aminotransferase (AST) and alanine aminotransferase (ALT) activities were determined using AST and ALT assay kits (Abcam) according to the manufacturer's protocols.

### 4.7 H&E Staining

To evaluate organ‐specific toxicity, 6‐week‐old female C57BL/6 mice were randomly assigned to receive intravenous injections of PBS (control), alb‐Mycobac (300 µg of Capsule‐deficient Mycobac), or t‐SMC (300 µg of Capsule‐deficient Mycobac, equivalent to 21.17 nmol of R848). 7 days postinjection, major organs, including the liver, spleen, kidney, lung, and heart, were harvested. The collected organs were fixed in 4% paraformaldehyde (PFA) for 48 h, sectioned, and stained with hematoxylin and eosin (H&E). Histological images were acquired using a slide scanner (ScanScope CS2, Sungkyunkwan University Cooperative Center for Research Facilities).

### 4.8 Cellular Uptake Assay

The t‐SMC and Original Mycobac were reacted with sulfo‐Cy5 NHS ester (Lumiprobe) at a volume ratio of 1:1 for 1 h at room temperature (RT). Following the reaction, the mixture was centrifuged at 10 000 × g, and the pellet was washed twice with PBS to remove the unreacted free dye. RAW264.7 cells were seeded at a density of 1 × 10^5^ cells per well in a 12‐well plate. After 24 h, t‐SMC‐Cy5 (40 µg of Capsule‐deficient Mycobac) or Original Mycobac‐Cy5 (1.3 × 10^6^ CFU) was added. Following incubation, the cells were harvested at 30/60/120/240 min after, and the intracellular Cy5 fluorescence intensity was analyzed by flow cytometry using a BD FACS Canto II (Sungkyunkwan University Cooperative Center for Research Facilities).

### 4.9 Biodistribution analysis

The alb‐Mycobac, t‐SMC, and Original Mycobac were reacted with sulfo‐Cy5 NHS ester (Lumiprobe) at a volume ratio of 1:1 for 1 h at RT. Following the reaction, the mixture was centrifuged at 10 000 × g, and the pellet was washed twice with PBS to remove the unreacted free dye. The resulting alb‐Mycobac‐Cy5 (300 µg of Capsule‐deficient Mycobac), t‐SMC‐Cy5 (300 µg of Capsule‐deficient Mycobac, equivalent to 21.17 nmol of R848), Original Mycobac‐Cy5 (1 × 10^7^ CFU) were administered intravenously. At 1, 24, 48, and 72 h postinjection, major organs (liver, spleen, kidney, lung, heart, and bone marrow) were harvested and fixed in 4% PFA. The distribution of t‐SMC‐Cy5 in each organ was analyzed with an IVIS Lumina XR optical imaging system (Perkin Elmer, Sungkyunkwan University Cooperative Center for Research Facilities) and Fobi (Neoscience).

### 4.10 Analysis of Splenic HSPCs population

A 6‐week‐old female C57BL/6 mice were randomly assigned to receive intravenous injections of PBS (control), alb‐Mycobac (300 µg of Capsule‐deficient Mycobac), or t‐SMC (300 µg of Capsule‐deficient Mycobac, equivalent to 21.17 nmol of R848). 7 days post‐injection, the spleens were mechanically disrupted, and red blood cells were removed using RBC lysis buffer (BioLegend). The resulting cell suspensions were filtered through 70 µm cell strainers and washed twice with PBS. Single cells from the tumors and spleen were stained with viability stain (BD) to segregate live cells and then stained with surface marker antibodies specific for LSK cells (Lineage^,^ Sca1, and c‐Kit), MPPs (CD150 and CD48), MPP3 (CD150 and Flt3), macrophages (CD11b, F4/80, CD80, and CD86) and monocytes (CD11b and Ly6C) for 30 min at 4°C. After staining, the cells were washed twice with Wash buffer and resuspended in 4% PFA. Flow cytometry data were acquired using a BD FACS Canto II (Sungkyunkwan University Cooperative Center for Research Facilities) and analyzed with FlowJo V10. Detailed gating strategy is provided in Figures S15 and S16.

### 4.11 Analysis of Splenic Macrophage Metabolism and Restimulation Assay

The F4/80^+^ splenic macrophages were isolated from splenocytes using UltraPure anti‐F4/80 MicroBeads, LS columns, and a QuadroMACS Separator (Miltenyi Biotec). After isolation, 1 × 10^6^ cells were harvested, and intracellular lactate levels were measured using an L‐Lactate Assay Kit (Abcam). For the restimulation assay, F4/80^+^ splenocytes from each group were seeded at 1 × 10^6^ cells per well in 12‐well plates and cultured in DMEM supplemented with 10% FBS and 1% streptomycin‐penicillin at 37°C for 24 h. LPS, a TLR4 ligand, was selected as the heterologous secondary stimulus because the heightened response to a heterologous secondary stimulus, primed by an unrelated PAMP (here, BCG‐derived components), constitutes the operational definition of TI [3, 35, 36, 37, 38, 39]. The cells were then treated with LPS (10 ng mL^−1^) for 24 h, after which the supernatants were collected for cytokine analysis.

### 4.12 ChIP‐qPCR

Isolated F4/80^+^ splenic macrophages were fixed in 1% PFA at room temperature for 15 min to crosslink the genomic DNA and proteins. To quench residual PFA, glycine (Sigma) was added to the crosslinked cells at a final concentration of 125 mM. Cells were then lysed using cell lysis buffer (Cell Signaling Technology) supplemented with a protease‐phosphatase inhibitor cocktail (Thermo). Chromatin was fragmented by sonication, and the lysate was clarified by centrifugation at 10 000 × g for 15 min at 4°C. For immunoprecipitation, an EZ‐Magna ChIP A/G kit (Sigma) was used. A 2 µL of immunoprecipitated DNA was used for qPCR in a 20 µL reaction volume. The amount of immunoprecipitated chromatin was calculated as a percentage of the input. Details of all primary antibodies and primer sequences used are provided in Tables S1 and S2.

### 4.13 Ex Vivo Splenocyte Restimulation Assay

A 6‐week‐old female C57BL/6 mice were randomly assigned to receive intravenous injections of PBS (control), Chol‐TLR7/8a liposome (21.17 nmol of R848) [18], alb‐Mycobac (300 µg of Capsule‐deficient Mycobac), or t‐SMC (300 µg of Capsule‐deficient Mycobac, equivalent to 21.17 nmol of R848). A 7 days post‐injection, splenocytes were harvested from each group, and 1 × 10^6^ cells were seeded per well in 12‐well plates. For the co‐culture model, 1 × 10^5^ MC38 or E.G7‐OVA cells were added to the splenocytes and co‐cultured in DMEM supplemented with 10% FBS and 1% streptomycin‐penicillin at 37°C for 6 h. For conditioned media, the splenocytes were cultured in 1 mL of MC38 or E.G7‐OVA conditioned media at 37°C for 6 h. For LPS restimulation model, cells were stimulated with LPS (10 ng mL^−1^) at 37°C for 6 h. The cells were then stimulated with Cell Activation Cocktail (with Brefeldin A, BioLegend) for 2 h before harvest to promote intracellular cytokine accumulation. After stimulation, the cells were harvested and stained with BD Horizon Fixable Viability Stain 780 and treated with surface marker antibodies specific for macrophages (CD11b and F4/80) and monocytes (CD11b and Ly6C) for 30 min at 4°C. For intracellular staining, the cells were washed and resuspended in fixation/permeabilization solution for 20 min at 4°C, then washed twice with BD Perm/Wash buffer (BD Bioscience) and treated with antibodies against cytokine‐producing macrophages or monocytes (TNF‐α) for 30 min at 4°C. After staining, the cells were washed twice with BD Perm/Wash buffer and resuspended in staining buffer. Detailed antibody information is provided in Table S1. Flow cytometry data were acquired using a BD FACS Canto II (Sungkyunkwan University Cooperative Center for Research Facilities) and analyzed with FlowJo V10. Detailed gating strategy is provided in Figure S17.

### 4.14 In Vitro BMDMs Restimulation Assay

BMDMs were generated from the bone marrow of 6‐week‐old female C57BL/6 mice. The femurs and tibias were isolated, and the bone marrow was flushed using a 26‐gauge syringe with DMEM (Gibco). To remove red blood cells, the marrow suspension was treated with RBC lysis buffer (BioLegend). Following multiple washes, the cells were resuspended in DMEM supplemented with 20 ng mL^−1^ recombinant mouse M‐CSF (mM‐CSF; R&D Systems) and seeded at a density of 1 × 10^7^ cells per 100 mm culture plate. Additional medium containing 20 ng mL^−1^ mM‐CSF was supplemented on day 3. On day 6, the differentiated immature BMDMs were harvested and seeded into 12‐well culture plates at a density of 1.5 × 10^5^ cells per well for further experiments. The cells were then stimulated with PBS (Control), Chol‐TLR7/8a with HSA (equivalent to 1.76 nmol R848), alb‐Mycobac (25 µg capsule‐deficient Mycobac), or t‐SMC (25 µg capsule‐deficient Mycobac and 1.76 nmol R848 equivalent). After a 24 h incubation, the cells were washed twice with PBS and incubated in fresh medium containing 20 ng mL^−1^ mM‐CSF. At 72 h post‐washing, the cells were restimulated with 10 ng mL^−1^ LPS for 24 h, after which the culture supernatants were collected for cytokine analysis.

### 4.15 Phagocytosis Assay

A 6‐week‐old female C57BL/6 mice were randomly assigned to receive intravenous injections of PBS (control), alb‐Mycobac (300 µg of Capsule‐deficient Mycobac), or t‐SMC (300 µg of Capsule‐deficient Mycobac, equivalent to 21.17 nmol of R848). A 7 days after injection, splenic macrophages were isolated from each group, and 1 × 10^6^ macrophages were seeded per well in 12‐well plates. Prior to incubation, E.G7‐OVA and MC38 cells were stained with 1 µM carboxyfluorescein succinimidyl ester (CFSE) for 1 h. For the coculture experiments, 1 × 10^5^ CFSE‐labeled MC38 or E.G7‐OVA cells were added to the splenic macrophages and incubated in DMEM supplemented with 10% FBS and 1% streptomycin‐penicillin at 37°C for 2 h. Following coculture, the cells were harvested, and the CFSE fluorescence intensity in the F4/80^+^ cells was analyzed by flow cytometry using a BD FACS Canto II (Sungkyunkwan University Cooperative Center for Research Facilities).

### 4.16 In Vivo Antitumor Study

A 6‐week‐old female C57BL/6 mouse received intravenous injections of PBS (control), alb‐Mycobac (300 µg of Capsule‐deficient Mycobac), or t‐SMC (300 µg of Capsule‐deficient Mycobac, equivalent to 21.17 nmol of R848). 7 days post‐injection, tumor models were established by subcutaneously injecting 5 × 10^5^ E.G7‐OVA cells into the right flank of each mouse. Tumor growth was monitored at various time points. Tumor volume was calculated using the following formula: long axis diameter × (short axis diameter)^2^/2. For immune cell population analysis, the tumors were mechanically dissociated and incubated in medium supplemented with collagenase D (1 mg mL^−1^; Sigma–Aldrich) at 37°C for 30 min in a shaking incubator. The spleens were also mechanically disrupted, and red blood cells were removed using RBC lysis buffer (BioLegend). The resulting cell suspensions were filtered through 70 µm cell strainers and washed twice with PBS. Single cells from the tumors and spleen were stained with viability stain (BD) to segregate live cells and then stained with surface marker antibodies specific for M1 macrophages (CD11b, F4/80, and CD80), M2 macrophages (CD11b, F4/80, and CD206), monocytes (CD11b and Ly6C), neutrophils (CD11b and Ly6g), dendritic cells (CD11c and CD86), CD8^+^ T cells (CD3 and CD8), and CD4^+^ T cells (CD3 and CD4). For intranuclear staining, the cells were washed and resuspended in fixation/permeabilization solution for 30 min at RT, then washed twice with eBioscience Foxp3/Transcription Factor Staining Buffer (Invitrogen) and stained with antibodies specific for T_h1_ cells (T‐bet) and T_reg_s (Foxp3). After staining, the cells were washed twice with Perm/Wash buffer and resuspended in staining buffer. Flow cytometry data were acquired using a BD FACS Canto II (Sungkyunkwan University Cooperative Center for Research Facilities) and analyzed with FlowJo V10. Detailed gating strategy is provided in Figures S16 and S18–S20. For CD8^+^ T cell depletion, mice received intraperitoneal injections of anti‐mouse CD8α at 100 µg per mouse on 8/10/12/14 days post‐inoculation.

### 4.17 RNA Sequencing

A 6‐week‐old female C57BL/6 mouse received intravenous injections of PBS (control) or t‐SMC (300 µg of Capsule‐deficient Mycobac, equivalent to 21.17 nmol of R848). 7 days following administration of PBS or t‐SMC, spleens were harvested, processed into a single‐cell suspension, and CD11b^+^ splenic myeloid cells were isolated from splenocytes using UltraPure Anti‐CD11b MicroBeads, LS columns, and a QuadroMACS Separator (Miltenyi Biotec). Samples were prepared in triplicate for each experimental group. Once these myeloid cells were isolated, RNAs were extracted using Trizol (Invitrogen). RNA quality was assessed by Agilent 4200 TapeStation System (Agilent Technologies), and RNA quantification was performed using Qubit (Thermo Fisher Scientific Inc.). For control and test RNAs, the construction of library was performed using QuantSeq 3' mRNASeq V2 Library Prep Kit FWD (Lexogen) according to the manufacturer's instructions. The library was amplified to add the complete adapter sequences required for cluster generation. The finished library was purified from PCR components. High‐throughput sequencing was performed as single‐end 100 bp sequencing using NextSeq 2000 (Illumina). Data mining and graphic visualization were performed using ExDEGA (Ebiogen). The RNA sequencing (RNA‐seq) reported in this article has been deposited in the National Center for Biotechnology Information (NCBI)’s Gene Expression Omnibus (GEO) (GSE319216). The RNA‐seq data (GSE319216) are currently in a private state. Reviewers can access the data using the following token: idqpekmufditpwh

### 4.18 Analysis of the Antigen‐Specific Immune Response

For Ag CD8^+^ T‐cell analysis, single splenocytes (5 × 10^5^ per well) were seeded in a round‐bottom 96‐well plate. The cells were subsequently restimulated with the OVA peptide SIINFEKL (2 µg) or tumor lysate (2 µg) together with GolgiPlug (a protein transport inhibitor, 0.6 µg mL^−1^; BD Bioscience) for 6 h. After stimulation, the cells were harvested and washed twice with PBS. Next, the cells were stained with BD Horizon Fixable Viability Stain 780 and surface marker antibodies specific for CD8^+^ T cells (CD3 and CD8) for 30 min at 4°C. After surface staining, the cells were subjected to intracellular staining. Briefly, the cells were washed and resuspended in fixation/permeabilization solution for 30 min at room temperature (RT) and then washed twice with eBioscience Intracellular Fixation & Permeabilization Buffer (Invitrogen). Thereafter, the cells were stained with antibodies specific for cytokine‐producing CD8^+^ T cells (IFN‐γ and TNF‐α) for 30 min at RT. Finally, the cells were washed twice with BD Perm/Wash buffer and resuspended in staining buffer. Flow cytometry data were acquired using a BD FACS Canto II (Sungkyunkwan University Cooperative Center for Research Facilities) and analyzed with FlowJo V10. Detailed gating strategy is provided in Figures S18 and S20.

### 4.19 Splenic Myeloid Cell Adoptive Transfer Model

A 6‐week‐old female C57BL/6 mouse received intravenous injections of either PBS (control) or t‐SMC (300 µg of Capsule‐deficient Mycobac, equivalent to 21.17 nmol of R848). A 7 days after injection, CD11b^+^ splenic myeloid cells were isolated from splenocytes using UltraPure Anti‐CD11b MicroBeads, LS columns, and a QuadroMACS Separator (Miltenyi Biotec) according to the manufacturer's instructions. Following isolation, 5 × 10^5^ CD11b^+^ cells were harvested and transferred into E.G7‐OVA tumor‐bearing mice via intratumoral injection. Adoptive transfer was performed four times at 2‐day intervals.

### 4.20 Surgical Model

On day 12 after the inoculation of 1 × 10^6^ MC38 cells, when the primary tumors had reached approximately 250–300 mm^3^ in size, approximately 90% of tumor removal was performed. The mice were anesthetized with isoflurane (HANA PHARM), the surgical site was sprayed with 70% ethanol, and approximately 90% of the tumor was excised, leaving 10% of the tumor tissue in place. To control tumor resection size: (i) Tumors were measured and randomly allocated for each group. (ii) Tumors were removed (approximately 90% of the primary tumor). (iii) Tumor removal was verified by measuring the resected tumor weight (Figure S22). For PD‐L1 expression analysis on recurrent tumors, tumor tissues were harvested 10 days post‐surgery, dissociated as described above, and analyzed by flow cytometry using PE/APC anti‐mouse CD274 (PD‐L1) antibody. Cells were gated on viable CD45^−^ tumor cells, and PD‐L1 expression was quantified as MFI. For the preoperative adjuvant model, MC38 tumor‐bearing mice received two intravenous injections of t‐SMC (300 µg of Capsule‐deficient Mycobac, equivalent to 21.17 nmol of R848) on days 6 and 4 prior to surgery. Anti‐mouse PD‐L1 antibody was administered intraperitoneally at 100 µg per mouse on 0/2/4/6/8 days post‐surgery (monotherapy/combination model). Oxaliplatin (MCE, HY‐17371) was reconstituted in D5W and administered intraperitoneally at 6 mg/kg on 0/5/10/15/20 days post‐surgery (monotherapy/combination model).

### 4.21 PBMCs Analysis

Isolation of ex vivo primary monocytes/macrophages from peripheral blood was performed using Histopaque‐1083 (Sigma). Then, 1 × 10^6^ cells were evenly distributed into 6‐well plates and stimulated with LPS (10 ng mL^−1^) at 37°C for 6 h. Subsequently, the cells were treated with Cell Activation Cocktail (containing Brefeldin A; BioLegend; Cat. No. 423303) for 2 h prior to being harvested. Following stimulation, the cells were collected, washed with PBS, and stained with BD Horizon Fixable Viability Stain 780, along with surface antibodies specific for myeloid cells (CD11b), macrophages (CD11b and F4/80), and monocytes (CD11b and Ly6C). For intracellular cytokine detection, the cells were fixed and permeabilized for 30 min at RT using eBioscience Intracellular Fixation & Permeabilization Buffer (Invitrogen), followed by two washes. The cells were then stained with an anti‐TNF‐α antibody. After two additional washes with BD Perm/Wash buffer, the cells were resuspended in staining buffer for analysis. Flow cytometry was performed using a BD FACS Canto II (Sungkyunkwan University Cooperative Center for Research Facilities), and data analysis was conducted with FlowJo V10. Detailed gating strategy is provided in Figure S21.

### 4.22 Statistical Analysis and Reproducibility

All the results, obtained from a minimum of three independent experiments, are presented as the mean ± standard deviation (SD). A two‐tailed unpaired *t* test was used to compare the two groups. One‐way ANOVA (or two‐way ANOVA) with Tukey's multiple comparisons test was used to analyse multiple groups of data. The Log‐rank (Mantel–Cox) test was used to analyze survival data. All the statistical analyses were performed with GraphPad Prism 8. *p* values (ns: not significant, *p < 0.05, **p < 0.01, ***p < 0.001, and ****p < 0.0001) were used to indicate statistical significance.

## Author Contributions

J.H.C. and Y.T.L. conceived and designed the trial. J.H.C., S.N.L., J.H.H., S.P., and S.H.P. performed the experiments, and J.H.C. analyzed the data. J.H.C. and Y.T.L. wrote the manuscript. Y.T.L. edited the manuscript and supervised the project. All the authors read and approved the final manuscript.

## Conflicts of Interest

The authors declare no conflicts of interest.

## Supporting information




**Supporting File**: adma74513‐sup‐0001‐SuppMat.pdf.

## Data Availability

All the data are included in the Supporting Information or available from the authors, as are the unique reagents used in this article.
