# Supplementary material for: Anisotropic Masked Mycobacterium Potentiates Amplified Antitumor Trained Immunity via Spleen Targeting and Myelopoiesis Conversion
Source: Adv Mater. 2026 Aug 5;38(56):e74513. doi: 10.1002/adma.74513 (PMC13648925; doi:10.1002/adma.74513)
Supplement: Supplementary file 1 [file ADMA-38-e74513-s001.pdf]

[Supporting Information]

Anisotropic Masked Mycobacterium Potentiates Amplified Antitumor Trained Immunity via Spleen Targeting and Myelopoiesis Conversion

Jin-Ho Choi, Sang Nam Lee, Jang Hun Heo, Soyoung Park, Sei Hyun Park, Yong Taik Lim*[1]

[1]SKKU Advanced Institute of Nanotechnology (SAINT), Department of Nano Science and Technology, Department of Nano Engineering, School of Chemical Engineering, and Biomedical Institute for Convergence at SKKU, Sungkyunkwan University, Suwon, Republic of Korea

***Corresponding author**

E-mail: yongtaik@skku.edu

Table of Contents

Table ·· **3**

Table S1 | List of antibodies.·· 3

Table S2 | List of primers used for ChIP-qPCR analysis.··································· 5

Figure ·· **6**

Figure S1 | Synthesis of Chol-R848.·· 6

Figure S2 | ^{1}H NMR and LC result of synthesized Chol-R848. ·························· 8

Figure S3 | GILT activable Chol-TLR7/8a analysis. ······································· 9

Figure S4 | Water dispersibility analysis of alb-Mycobac, t-SMC, and Original Mycobac.··· 10

Figure S5 | Evaluation of the immunological effects of t-SMC.····························· 11

Figure S6 | Biodistribution analysis of t-SMC and Original Mycobac.······················ 12

Figure S7 | Assessment of t-SMC biocompatibility. ·· 13

Figure S8 | Increased M1-like splenic macrophage by t-SMC. ······························ 14

Figure S9 | Trained immunity against tumor by t-SMC.····································· 15

Figure S10 | BMDMs *ex vivo* restimulation assay. ·· 16

Figure S11 | Innate immune population analysis of TI-mediated anti-tumor model by t-SMC.
·· 17

Figure S12 | Transcriptional analysis of trained immunity effect on splenic myeloid cell induced by t-SMC.··· 18

Figure S13 | Effects of adaptive immune cell populations on t-SMC-based trained immunity anti-tumor efficacy.·· 19

Figure S14 | Splenic antigen specific $CD8^{+}$ T cell and memory $CD8^{+}$ T cell analysis from operated mice.··· 20

Figure S15 | Gating strategies for analysis splenic HSPCs. ································ 21

Figure S16 | Gating strategies for analysis splenic myeloid cell population. ················ 22

Figure S17 | Gating strategies for analysis TNF-α$^+$ splenic myeloid cell population. ·········· 23

Figure S18 | Gating strategies for analysis splenic adaptive immune cell population. ········· 24

Figure S19 | Gating strategies for innate immune cell population in TME. ······················ 25

Figure S20 | Gating strategies for adaptive immune cell population in TME. ···················· 26

Figure S21 | Gating strategies for analysis TNF-α$^+$ myeloid cell population in PBMCs. ······ 27

Figure S22 | Tumor resection procedure. ··· 28

Antibody	Manufacturer	Clone	Catalog number
Flow cytometry			
FITC anti-mouse Lineage Cocktail with Isotype Ctrl	Biolegend	145-2C11; RB6-8C5; RA3-6B2; Ter-119; M1/70;	133302
APC anti-mouse CD117 (c-Kit)	Biolegend	2B8	105812
APC/Cyanine7 anti-mouse Ly-6A/E (Sca-1)	Biolegend	D7	108126
PerCP/Cyanine5.5 anti-mouse CD48	Biolegend	HM48-1	103422
Brilliant Violet 421™ anti-mouse CD150 (SLAM)	Biolegend	TC15-12F12.2	115926
PE anti-mouse CD135	Biolegend	A2F10	135306
BD Horizon™ Fixable Viability Stain 780	BD		565388
Brilliant Violet 421™ anti-mouse/human CD11b Antibody	Biolegend	M1/70	101236
PerCP anti-mouse Ly-6G	Biolegend	1A8	127654
APC anti-mouse Ly-6C	Biolegend	HK1.4	128016
FITC anti-mouse F4/80	Biolegend	BM8	123108
APC anti-mouse F4/80	Biolegend	BM8	123116
PE anti-mouse CD11c	Biolegend	N418	117308
Brilliant Violet 421™ anti-mouse CD86	Biolegend	GL-1	105032
FITC anti-mouse CD80	Biolegend	16-10A1	104706
Brilliant Violet 421™ anti-mouse/human CD11b	Biolegend	M1/70	101236
PerCP/Cyanine5.5 anti-mouse TNF-α	Biolegend	MP6-XT22	506322
APC anti-mouse Ly-6G	Biolegend	1A8	127614
PE anti-mouse Ly-6C	Biolegend	HK1.4	128008
APC anti-mouse CD8a	Biolegend	53-6.7	100712
Brilliant Violet 421™ anti-mouse CD3	Biolegend	17A2	100228
FITC anti-mouse CD4	Biolegend	GK1.5	100406

PE anti-mouse IFN-γ	Biolegend	W18272D	163504
PE anti-mouse FOXP3	Biolegend	MF-14	126404
PerCP/Cyanine5.5 anti-T-bet	Biolegend	4B10	644806
PE anti-mouse CD62L	Biolegend	MEL-14	104408
FITC anti-mouse CD44	Biolegend	NIM-R8	156008
In vivo			
InVivo MAb anti-mouse PD-L1	Bio X cell	10F.9G2™	BE0101
*InVivo*MAb anti-mouse CD8α	Bio X cell	2.43	BE0061
Magnetic bead antibody for sorting			
Anti-F4/80 MicroBeads UltraPure, mouse	Miltenyi Biotec		130-110-443
CD11b MicroBeads, human and mouse	Miltenyi Biotec		130-049-601
ChIP-qPCR			
Tri-Methyl-Histone H3 (Lys4) (C42D8) Rabbit Monoclonal Antibody	Cell Signalling		#9751
CLSM			
Human Serum Albumin Alexa Fluor® 488-conjugated Antibody	R&D Systems		IC1455G

Table S1. List of antibodies.

Primer	Forward	Reverse
ChIP-qPCR		
Tnf promoter	CACCCCGAAGTTCAGTAGACA	GAACTGGCAGAAGAGGCACT
Il6 promoter	TTTCTCCACGCAGGAGACTT	TCCACGATTTCCCAGAGAAC

Table S2. List of primers used for ChIP-qPCR analysis.

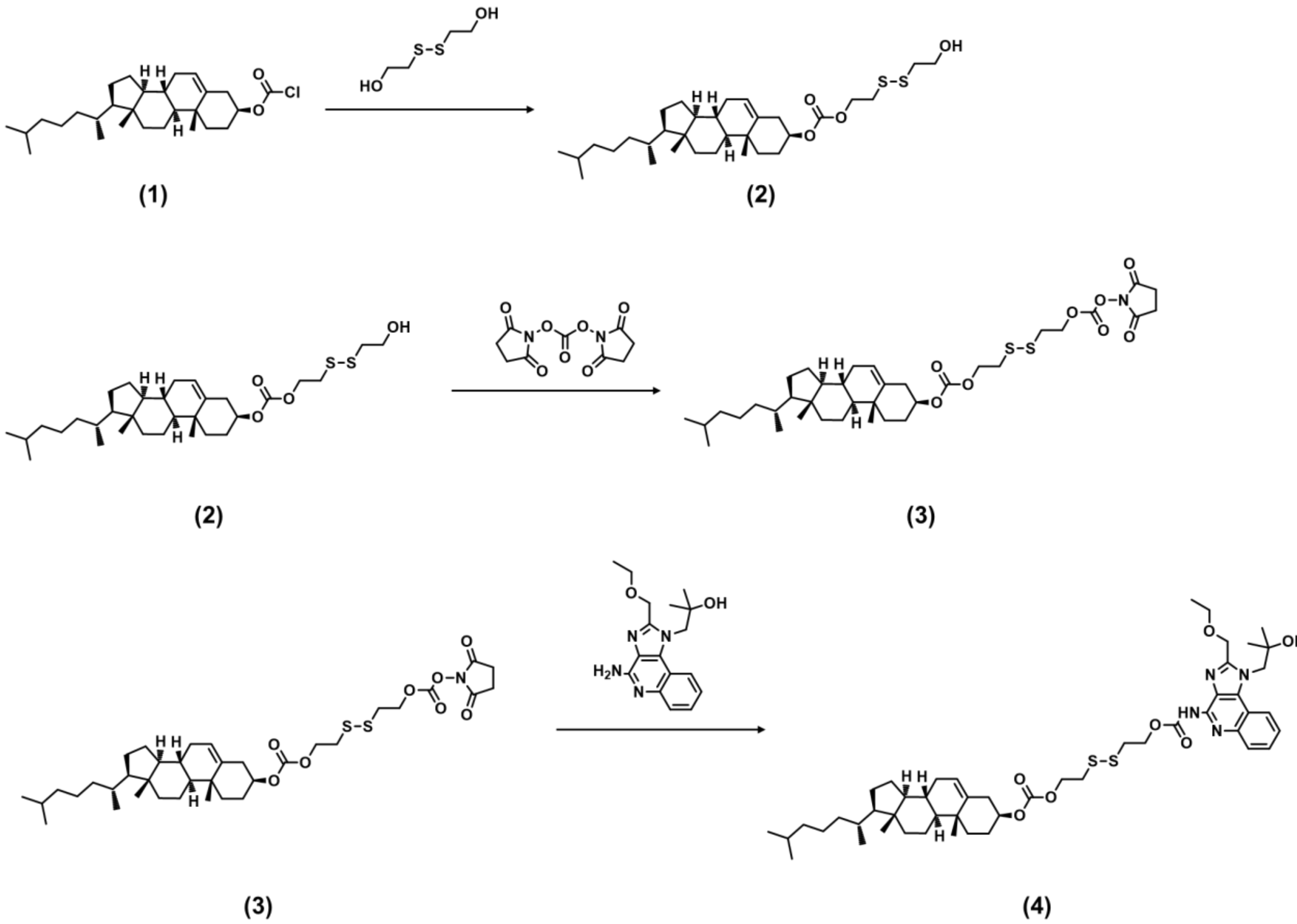

Figure S1. Synthesis of Chol-TLR7/8a. Cholesteryl chloroformate (compound (1), 40 g) and DCM (100 ml) were added to the reactor at 10 ~ 15 °C. Then, a solution of bis(2-hydroxyethyl) disulfide in DCM (250 ml) and pyridine (21 g) were added to the mixture. The mixture was stirred at room temperature (RT) for 2 h. After that time, H2O was added, and DCM was used to extract the mixture. The combined organic layer was washed with brine (300 ml) and dried over anhydrous Na2SO4 (1 kg). (Compound (2) proton nuclear magnetic resonance 1H NMR, Bruker Avance III 700 MHz, CDCl3: δ 5.41-5.42 (m, 1H), 4.46-4.56 (m, 1H), 4.41 (t, J = 6.8 Hz, 2H), 3.91 (t, J = 6.0 Hz, 2H), 2.98 (t, J = 6.8 Hz, 2H), 2.91 (t, J = 6.0 Hz, 2H), 2.35-2.47 (m, 2H), 1.79-2.08 (m, 6H), 1.43-1.73 (m, 7H), 1.25-1.42 (m, 5H), 1.06-1.22 (m, 7H), 0.97-1.03 (m, 5H), 0.93 (d, J = 6.4 Hz, 3H), 0.88 (dd, J = 1.6, 6.8 Hz, 6H), and 0.69 (s, 3H)) Compound (2) (20 g) and DCM (200 ml) were added to the reactor. Additionally, bis(2,5-dioxopyrrolidin-1-yl) carbonate (18 g) and TEA (10.7 g) were added. The mixture was stirred at RT for 3 h. H2O was added, and DCM was used to extract the mixture. Then, the combined organic layer was washed with brine (200 ml) and dried over anhydrous Na2SO4 (20 g). R848 and DCM (1:13.26, w/w) were added to the reactor at 10 ~ 20 °C. (Compound (3) proton nuclear magnetic resonance 1H NMR, Bruker Avance III 700 MHz, CDCl3: δ 5.39-5.40 (m, 1H), 4.57 (t, J = 6.8 Hz, 2H), 4.43-4.52 (m, 1H), 4.37 (t, J = 6.4 Hz, 2H), 2.96-3.03 (m, 4H), 2.84 (s, 4H), 2.34-2.44 (m, 2H), 1.78-2.04 (m, 5H), 1.42-1.73 (m, 7H), 1.22-1.40 (m, 5H), 1.06-1.20 (m, 7H),

0.95-1.04 (m, 5H), 0.91 (d, J = 6.0 Hz, 3H), 0.86 (d, J = 6.4 Hz, 6H), and 0.67 (s, 3H)) Then, compound (3) (R848: compound (3) = 1:2.7, w/w) and TEA were added. The mixture was stirred overnight at 20 ~ 25 °C. H2O was added, and the mixture was extracted with DCM. The combined organic layer was washed with brine and dried over anhydrous Na2SO4. After filtration and drying under reduced pressure, the residue was purified by silica gel column chromatography, including elution with 10% ~ 50% EtOAc in n-hexane, yielding 10.8 g of compound (4) as a white solid (yield: 37.4%)

a

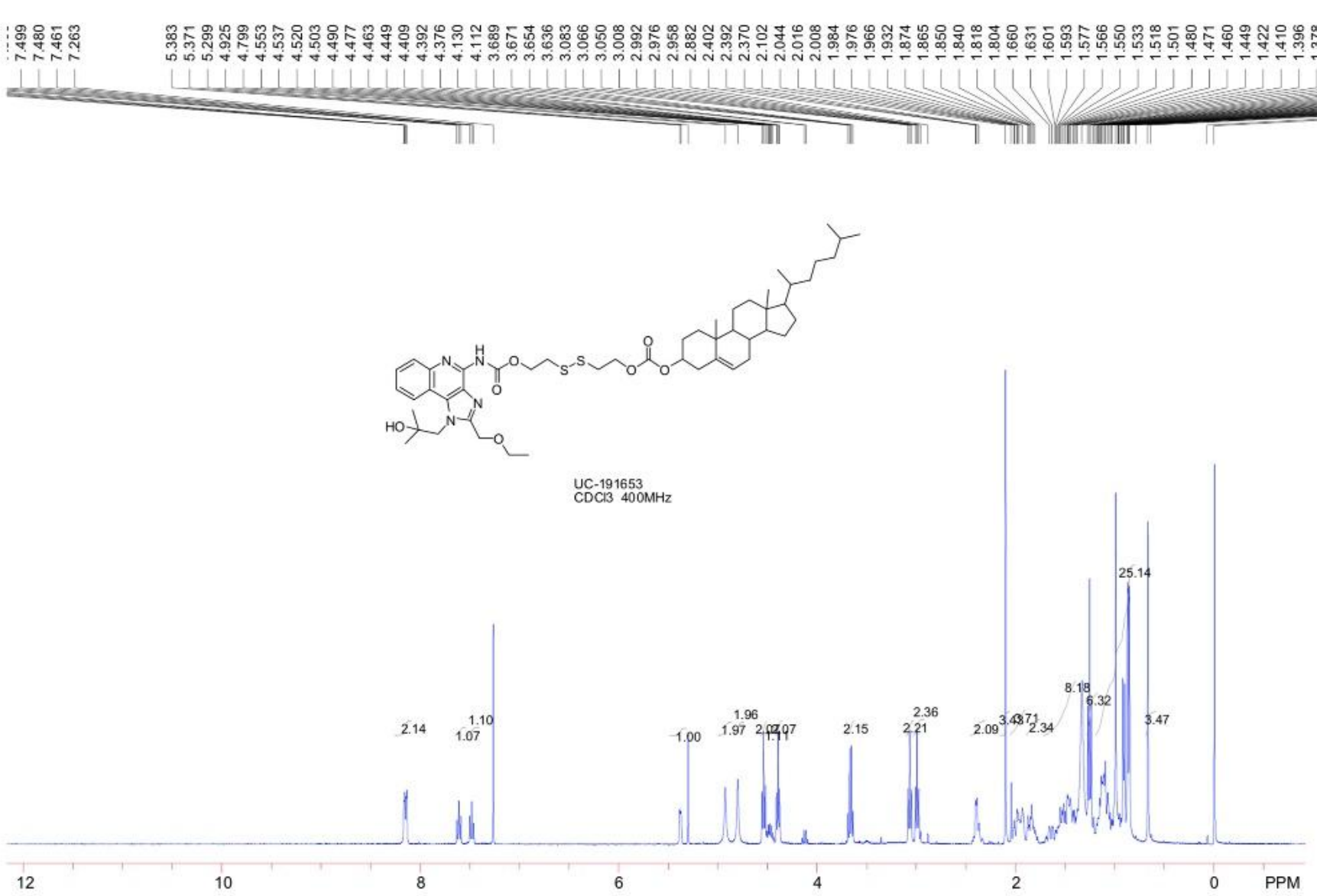

b

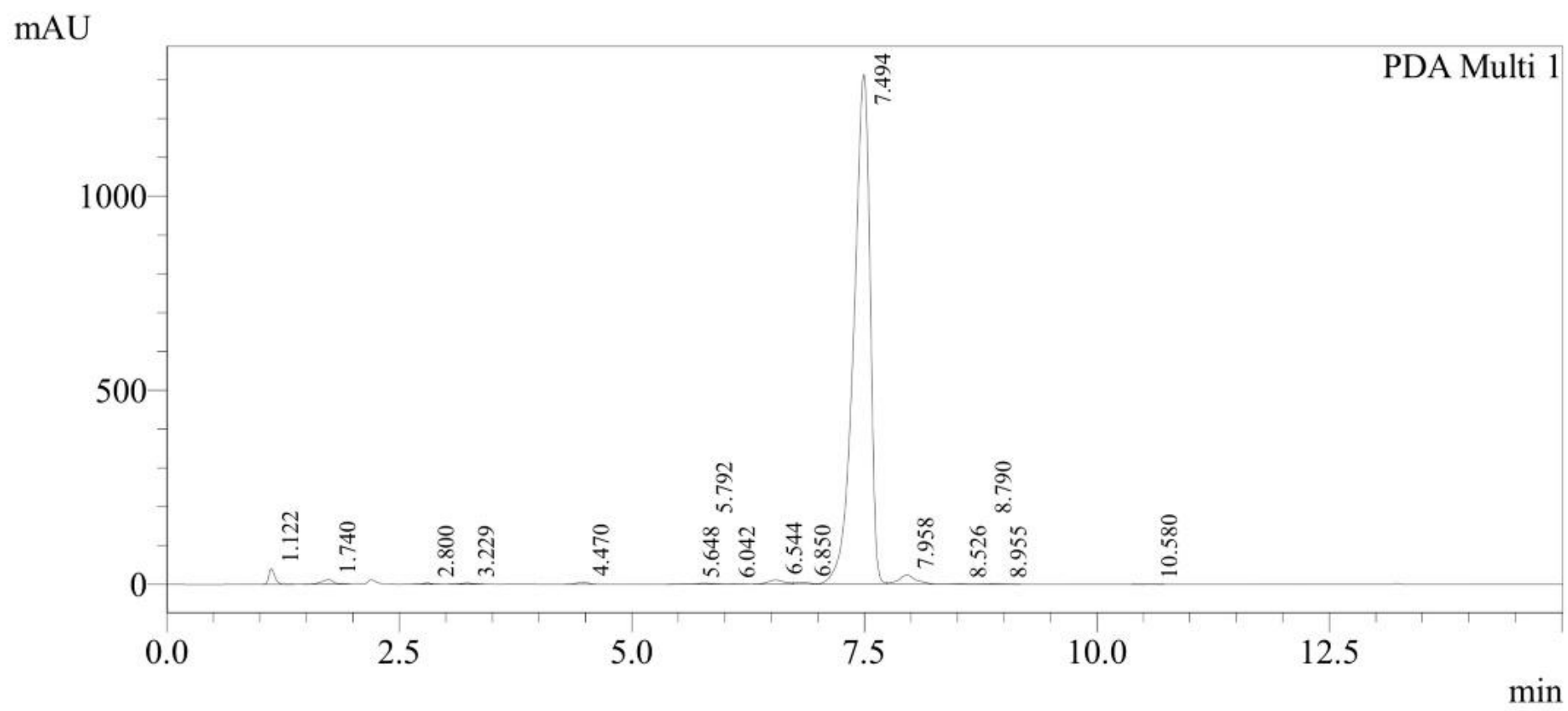

Figure S2. ¹H NMR and LC result of synthesized Chol-TLR7/8a. a) ¹H NMR (Bruker Avance Ⅲ 700 MHz, CDCl3): δ 5.39-5.40 (m, 1H), 4.57 (t, J = 6.8 Hz, 2H), 4.43-4.52 (m, 1H), 4.37 (t, J = 6.4 Hz, 2H), 2.96-3.03 (m, 4H), 2.84 (s, 4H), 2.34-2.44 (m, 2H), 1.78-2.04 (m, 5H), 1.42-1.73 (m, 7H), 1.22-1.40 (m, 5H), 1.06-1.20 (m, 7H), 0.95-1.04 (m, 5H), 0.91 (d, J = 6.0 Hz, 3H), 0.86 (d, J = 6.4 Hz, 6H), and 0.67 (s, 3H). b) Synthesized Chol-TLR7/8a was characterized by liquid chromatography (LC) using a mobile phase 0.05%TFA in water and ACN with C18 column.

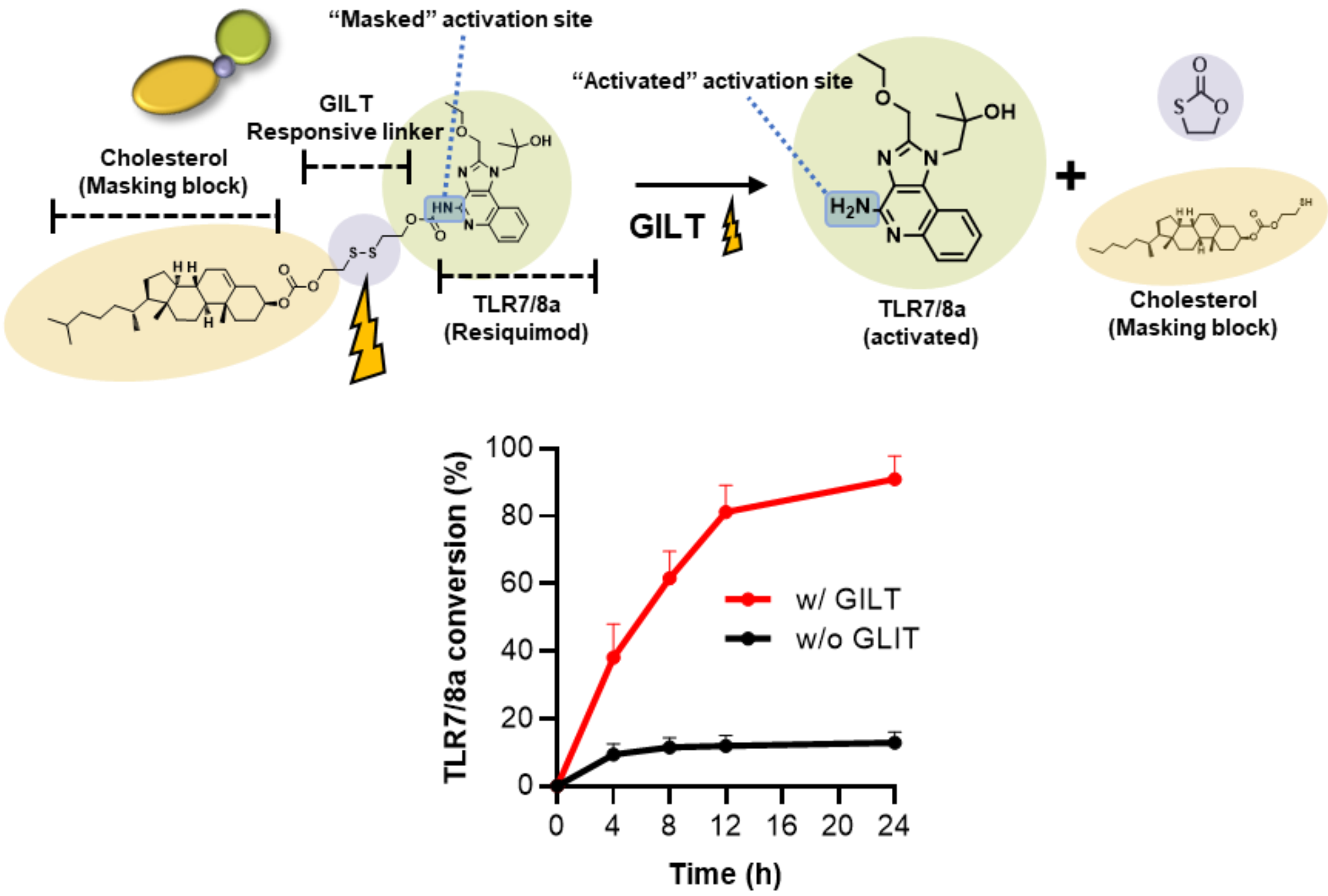

Figure S3. GILT activable Chol-TLR7/8a analysis. GILT-dependent TLR7/8a recovery kinetics of Chol-TLR7/8a

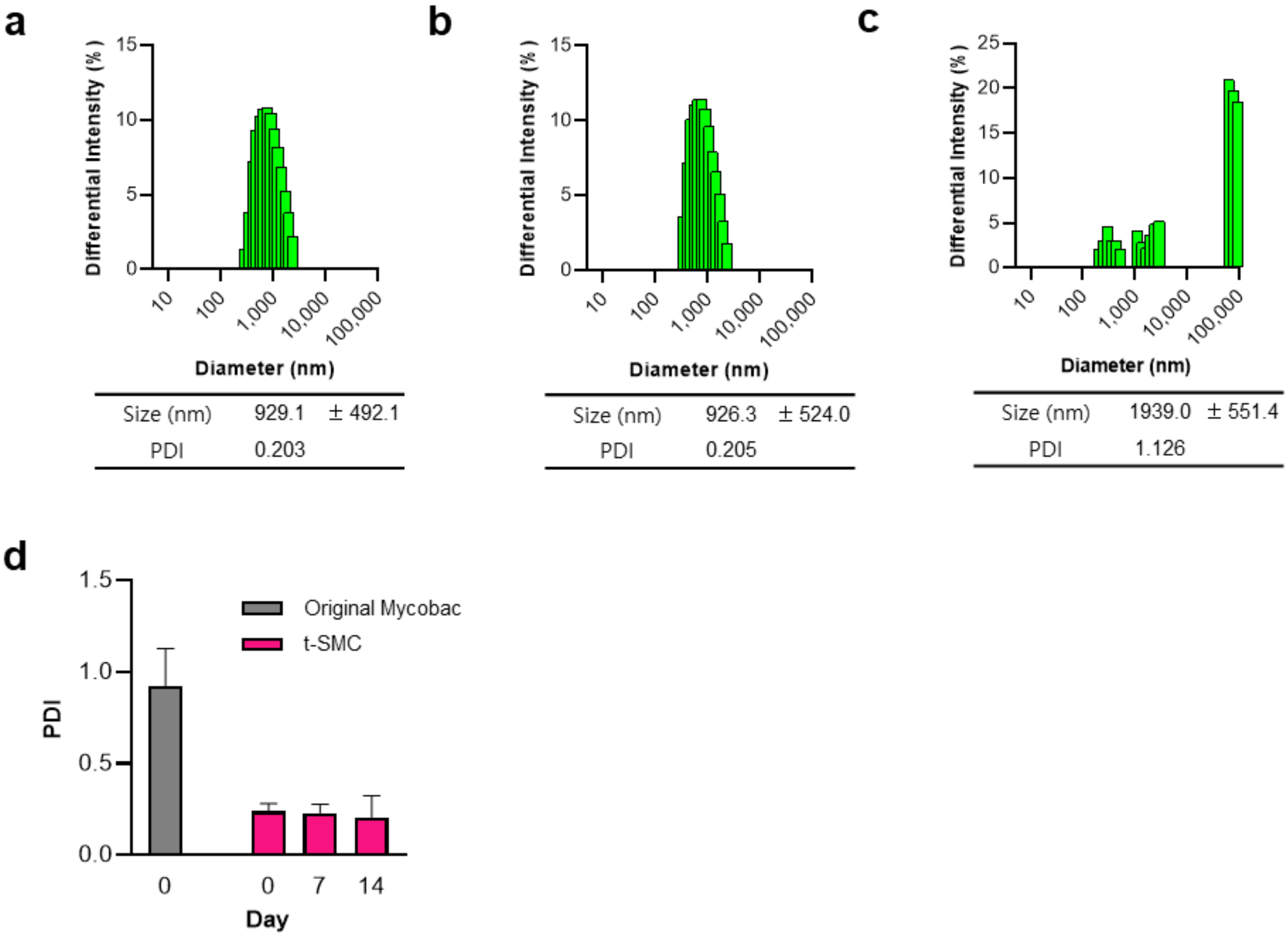

Figure S4. Water dispersibility analysis of alb-Mycobac, t-SMC, and Original Mycobac. a-c) Size distribution analysis of (a) alb-Mycobac, (b) t-SMC, and (c) Original Mycobac by dynamic light scattering (DLS). d) PDI analysis of t-SMC (4 °C, in PBS,14 days).

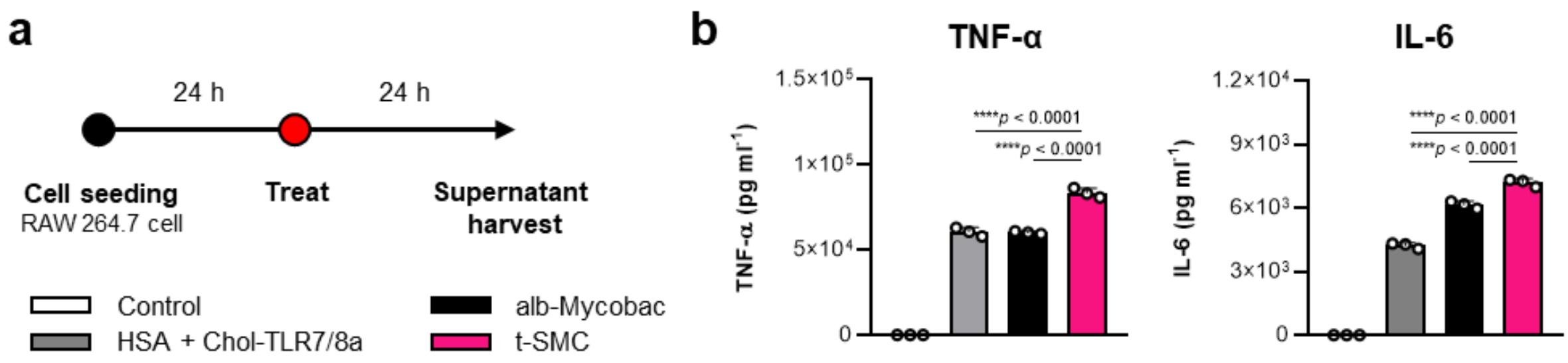

Figure S5. Evaluation of the immunological effects of t-SMC. a) Experimental scheme for immune cell activation analysis (n=3 per group). b) Cytokine secretion profiles in RAW264.7 cells 24 h after treatment with the indicated materials. All data are presented as the mean ± s.d. Statistical significance was evaluated by one-way ANOVA with Tukey's multiple-comparison test in b. *p*-values: $^{****}p < 0.0001$.

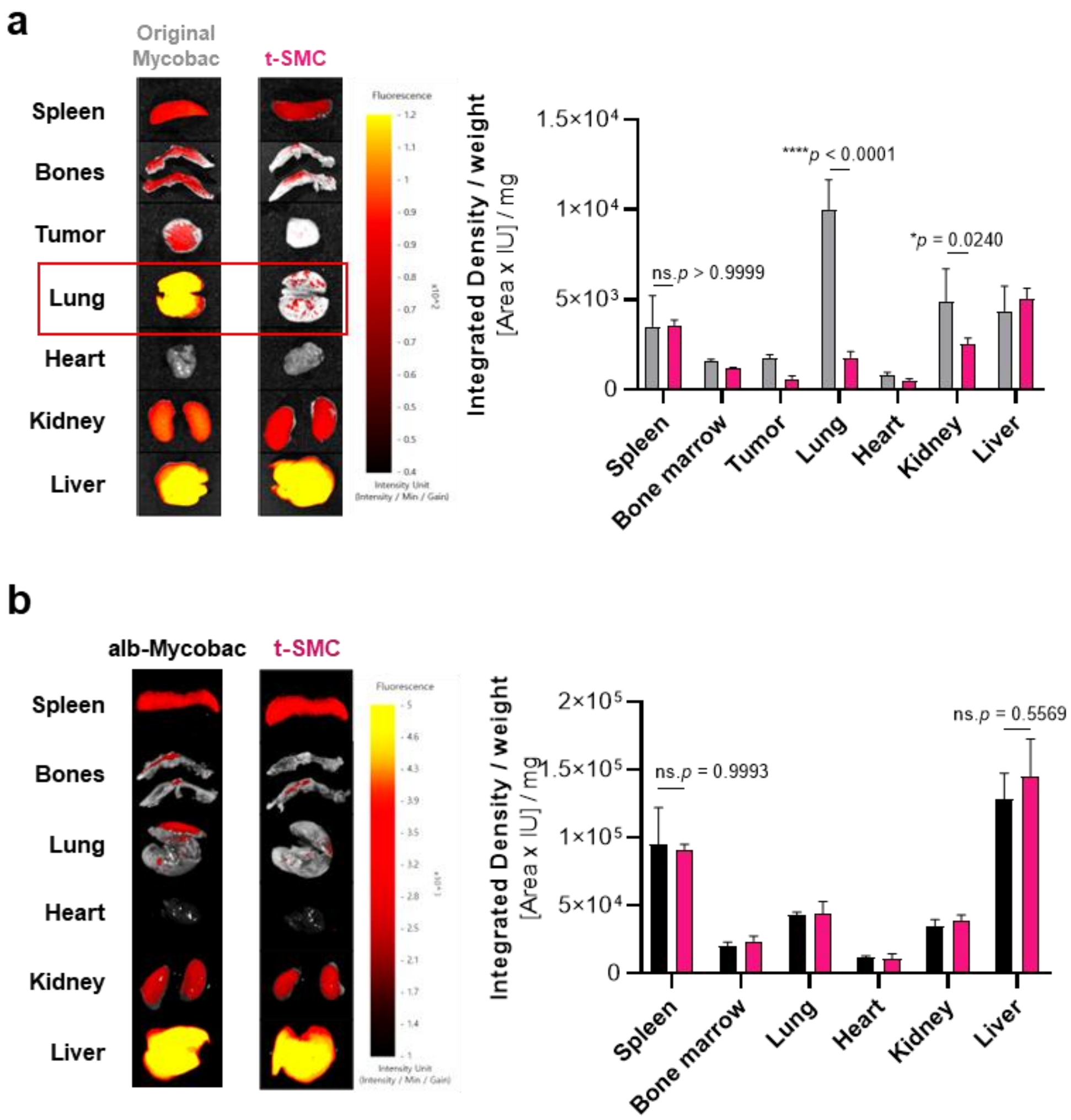

Figure S6. Biodistribution analysis of t-SMC and Original Mycobac. a) Biodistribution analysis of t-SMC and Original Mycobac 24 h after IV injection. b) Biodistribution analysis of alb-Mycobac and t-SMC 24 h after IV injection. All data are presented as the mean ± s.d. Statistical significance was evaluated by two-way ANOVA with Tukey's multiple-comparison test. *p*-values: ns, not significant; $*p < 0.05$; $****p < 0.0001$.

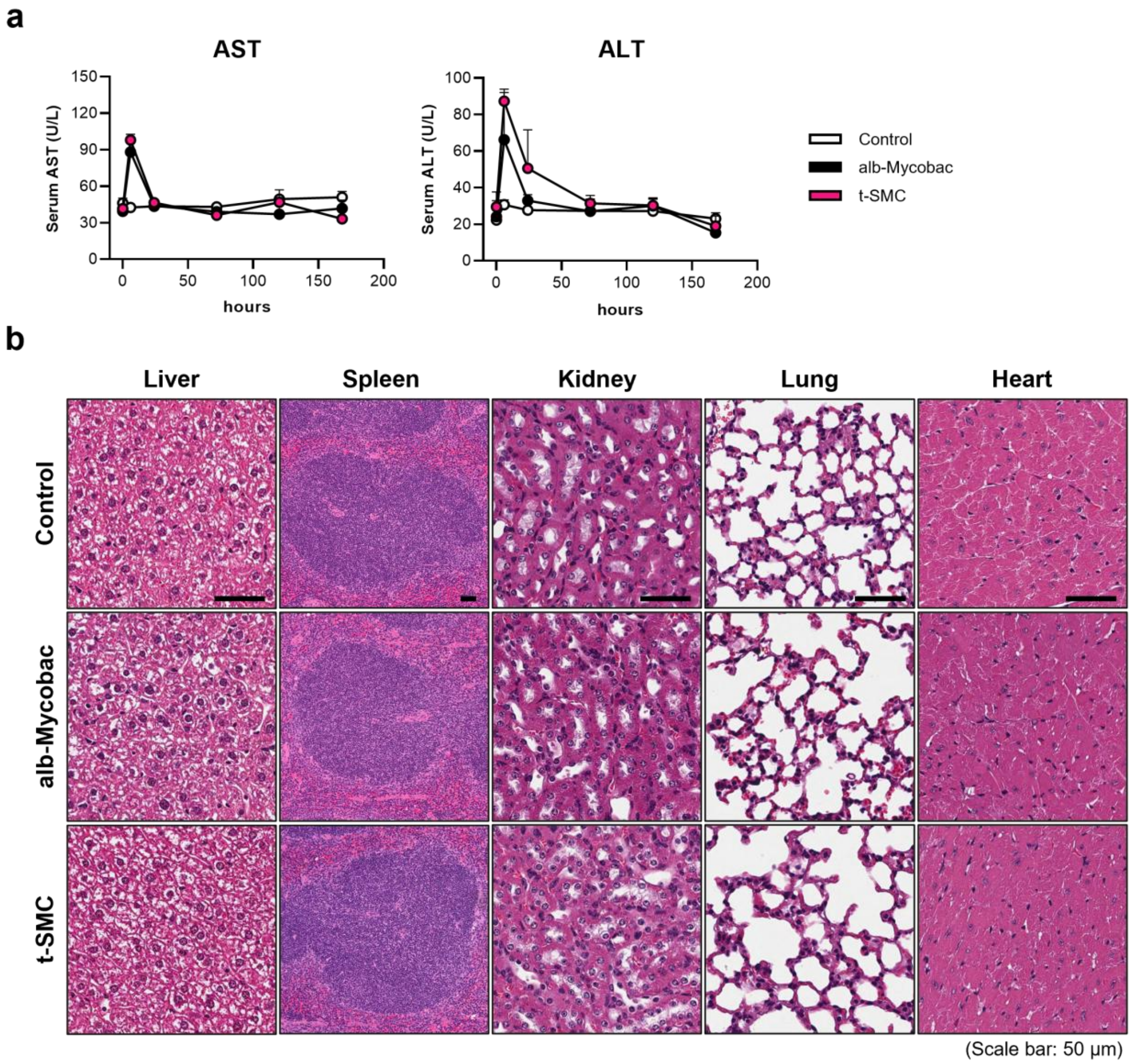

Figure S7. Assessment of t-SMC biocompatibility. a) Evaluation of liver toxicity by measuring serum aspartate aminotransferase (AST) and alanine aminotransferase (ALT) levels after injection. b) Comparative toxicity analysis of major organs analyzed by H&E staining 7 days after injection (scale bar: 50 μm). All the data are presented as the mean ± s.d.

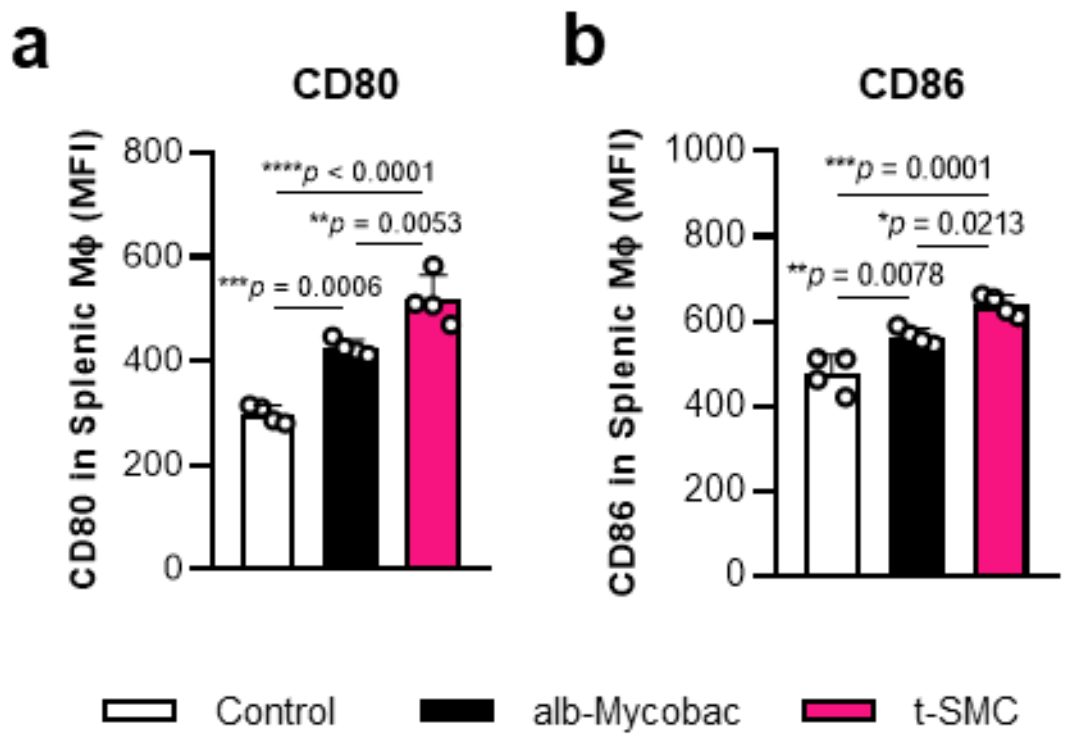

Figure S8. Increased M1-like splenic macrophage by t-SMC. a,b) Co-stimulatory receptor (a) CD80, and (b) CD86 analysis of splenic macrophage. All the data are presented as the mean ± s.d. Statistical significance was evaluated by one-way ANOVA with Tukey's multiple-comparison test in a,b. *p* values: **p* < 0.05; ***p* < 0.01; ****p* < 0.001; *****p* < 0.0001.

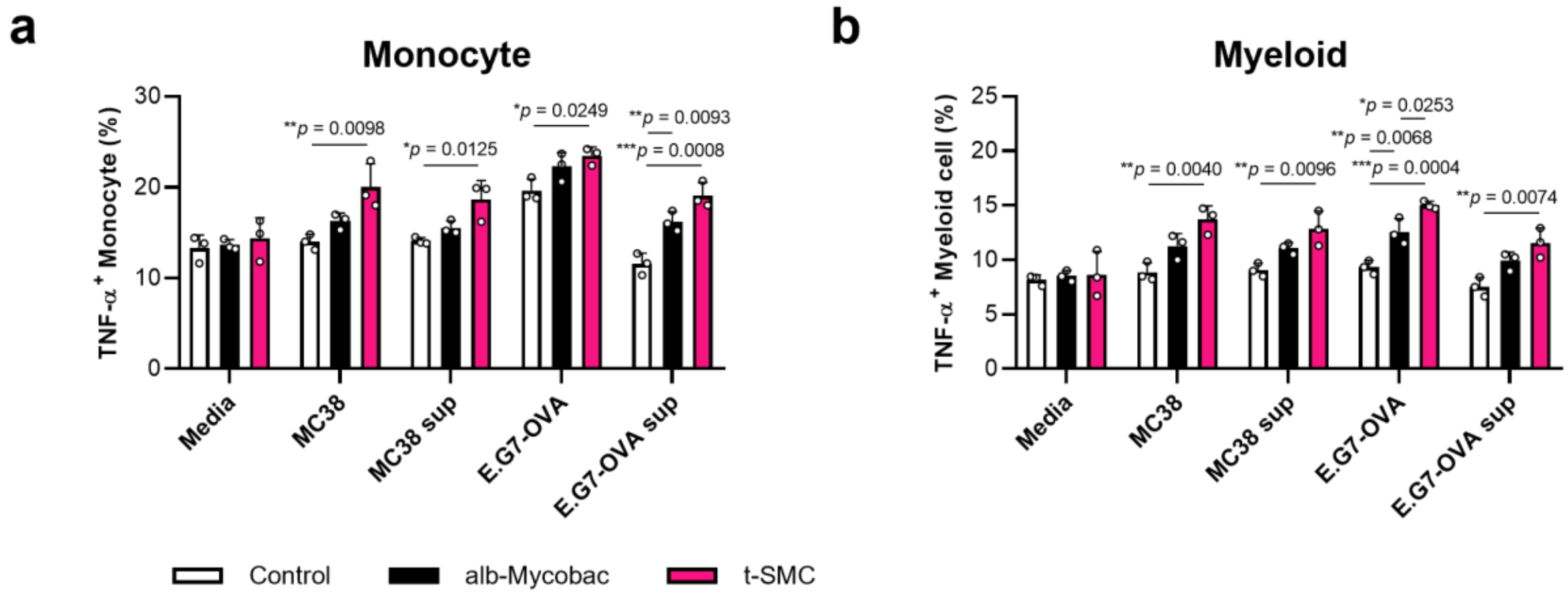

Figure S9. Trained immunity against tumor by t-SMC. a,b) Analysis of TNF-α positive (a) monocytes and (b) myeloid cells after coculture with tumor cells (E.G7-OVA or MC38) or the tumor cell supernatant. All the data are presented as the mean ± s.d. Statistical significance was evaluated by one-way ANOVA with Tukey's multiple-comparison test in a,b. *p* values: *$p < 0.05$; **$p < 0.01$; ***$p < 0.001$.

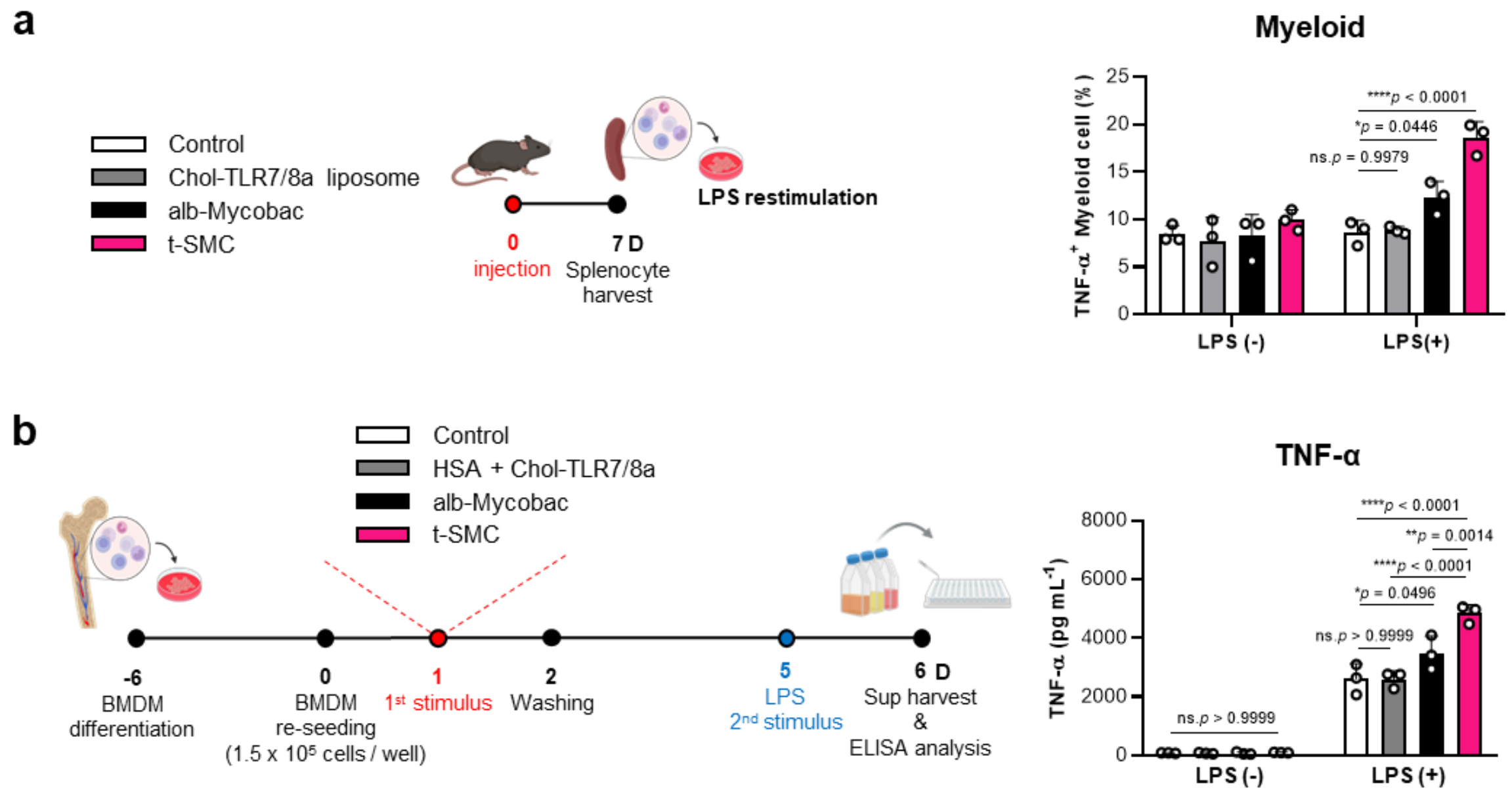

Figure S10. ***ex vivo, in vitro rest*****imulation assay.** a) 7 days after IV injection, splenocytes were restimulated with LPS and TNF-α$^+$ myeloid cells were analyzed (n=3 per group). b) BMDMs were trained and restimulated with LPS. Cytokine (TNF-α) secretion after LPS restimulation was measured (n=3 per group). All the data are presented as the mean ± s.d. Statistical significance was evaluated by one-way ANOVA with Tukey's multiple-comparison test in a and b. p-values: ns, not significant *p < 0.05; **p < 0.01; ****p < 0.0001.

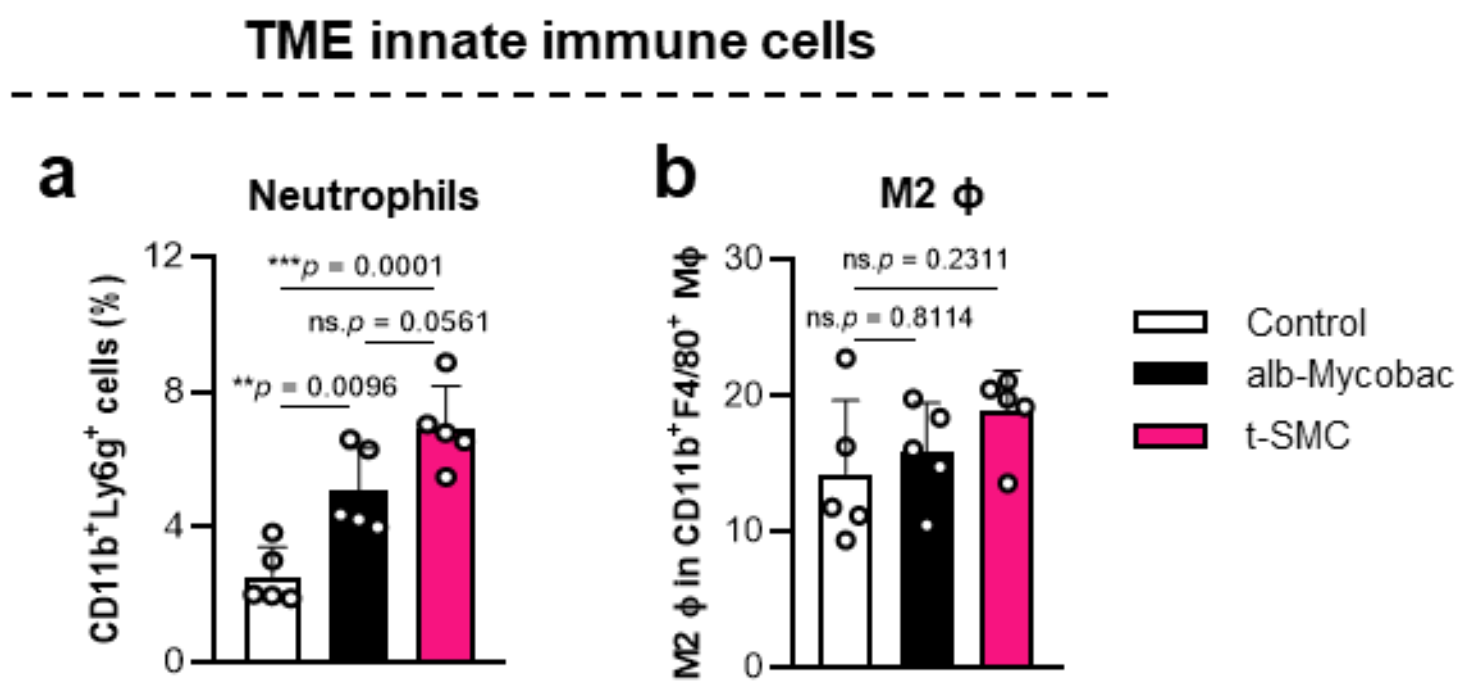

Figure S11. Innate immune population analysis of TI-mediated anti-tumor model by t-SMC. a,b) Analysis of innate immune cell populations in the TME : (a) neutrophils and (b) M2 macrophages. All data are presented as the mean ± s.d. Statistical significance was evaluated by one-way ANOVA with Tukey's multiple-comparison test. *p*-values: ns, not significant; $^{**}p < 0.01$; $^{***}p < 0.001$.

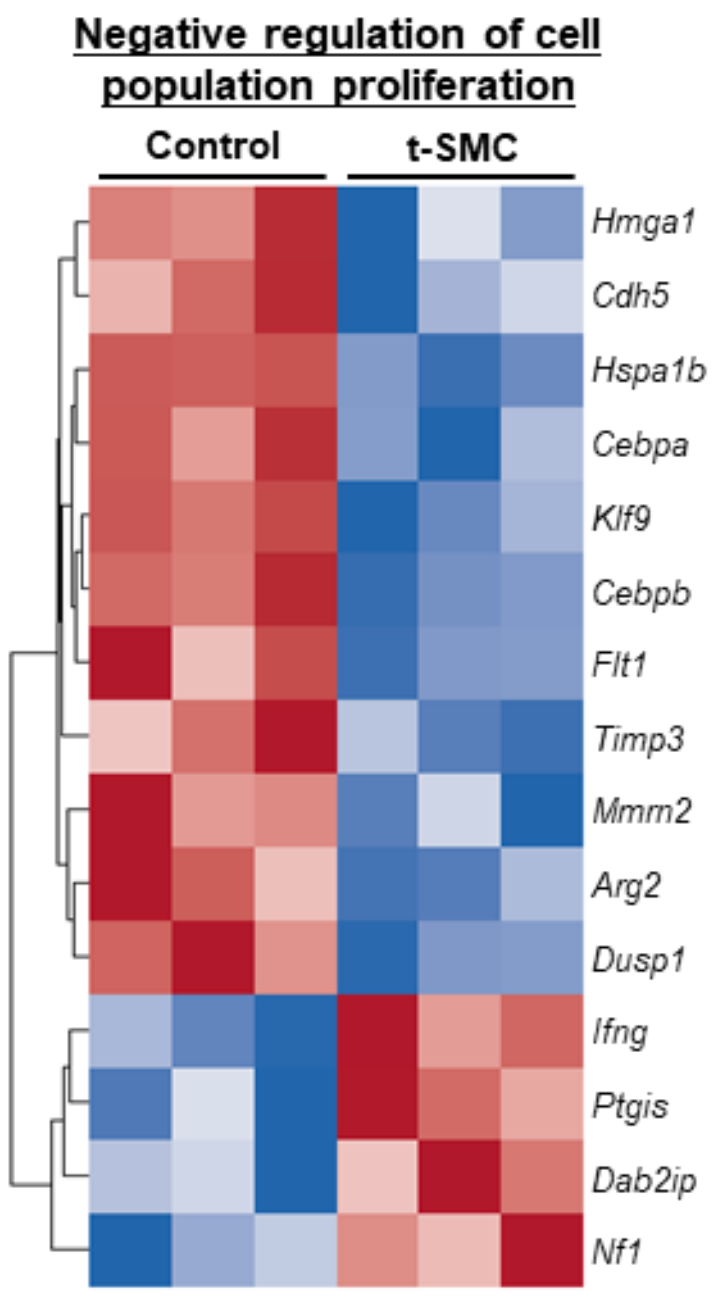

Figure S12. Transcriptional analysis of trained immunity effect on splenic myeloid cell induced by t-SMC. Heatmap of genes related to negative regulation of cell population proliferation.

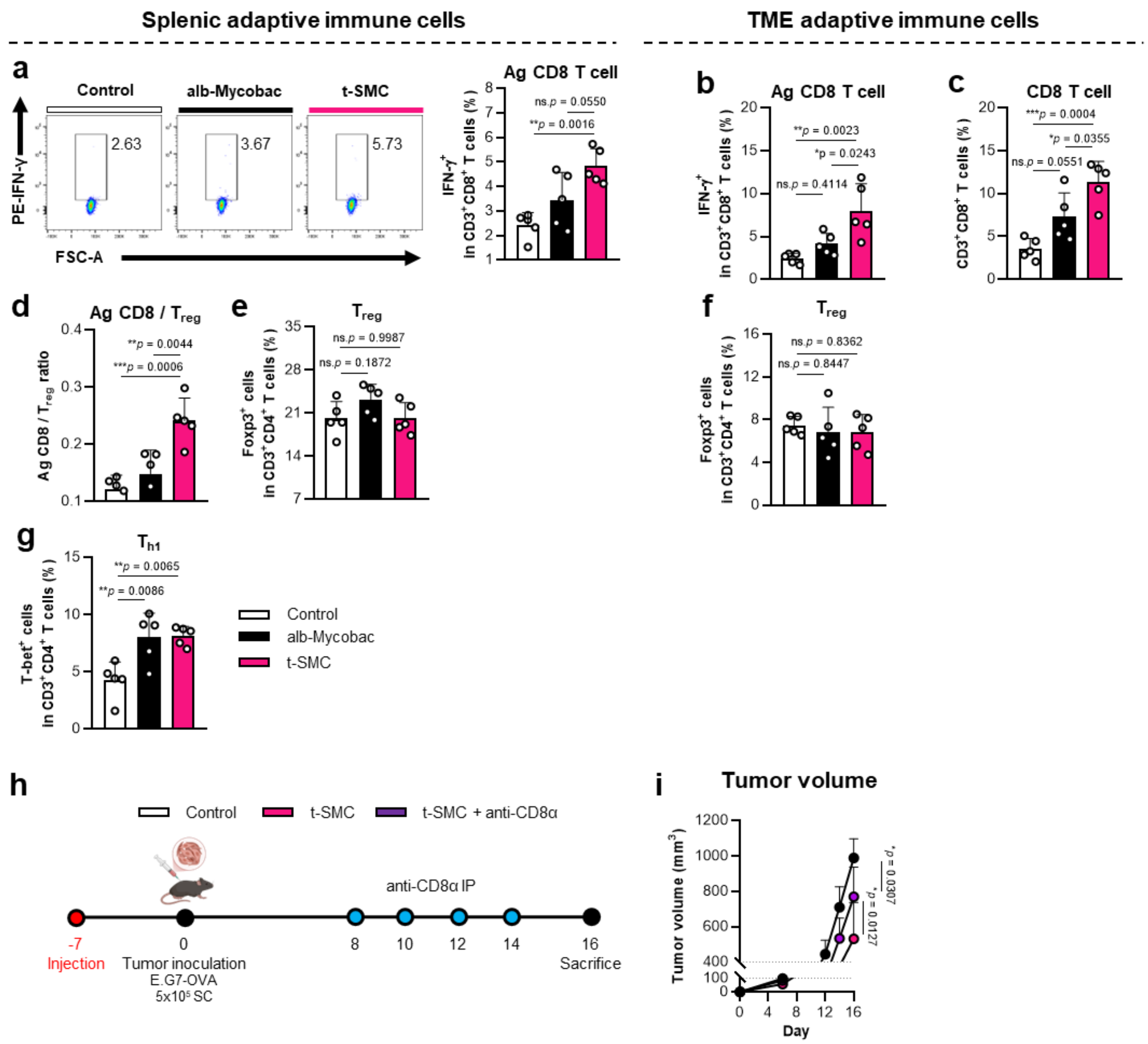

Figure S13. Effects of adaptive immune cell populations on t-SMC-based trained immunity anti-tumor efficacy. a-i) Assessment of adaptive immune cells in spleen and TME: (a) antigen-specific $CD8^+$ T cells in spleen, (b) antigen-specific $CD8^+$ T cells in TME, (c) $CD8^+$ T cell population in TME, (d) ratio of antigen-specific $CD8^+$ T cells to regulatory T cells (T_{reg}) in spleen, (e) T_{reg} population in spleen, (f) T_{reg} population in TME, and (g) type 1 helper T cells (T_{h1}) population in spleen. h) treatment schedule of $CD8^+$ T cell depletion analysis (n=4 per group). i) whole tumor volume of each group. All data are presented as the mean ± s.d. Statistical significance was evaluated by one-way ANOVA with Tukey's multiple-comparison test in a-g and two-way ANOVA with Tukey's multiple-comparison test in i. *p*-values: ns, not significant; $*p < 0.05$; $**p < 0.01$; $***p < 0.001$.

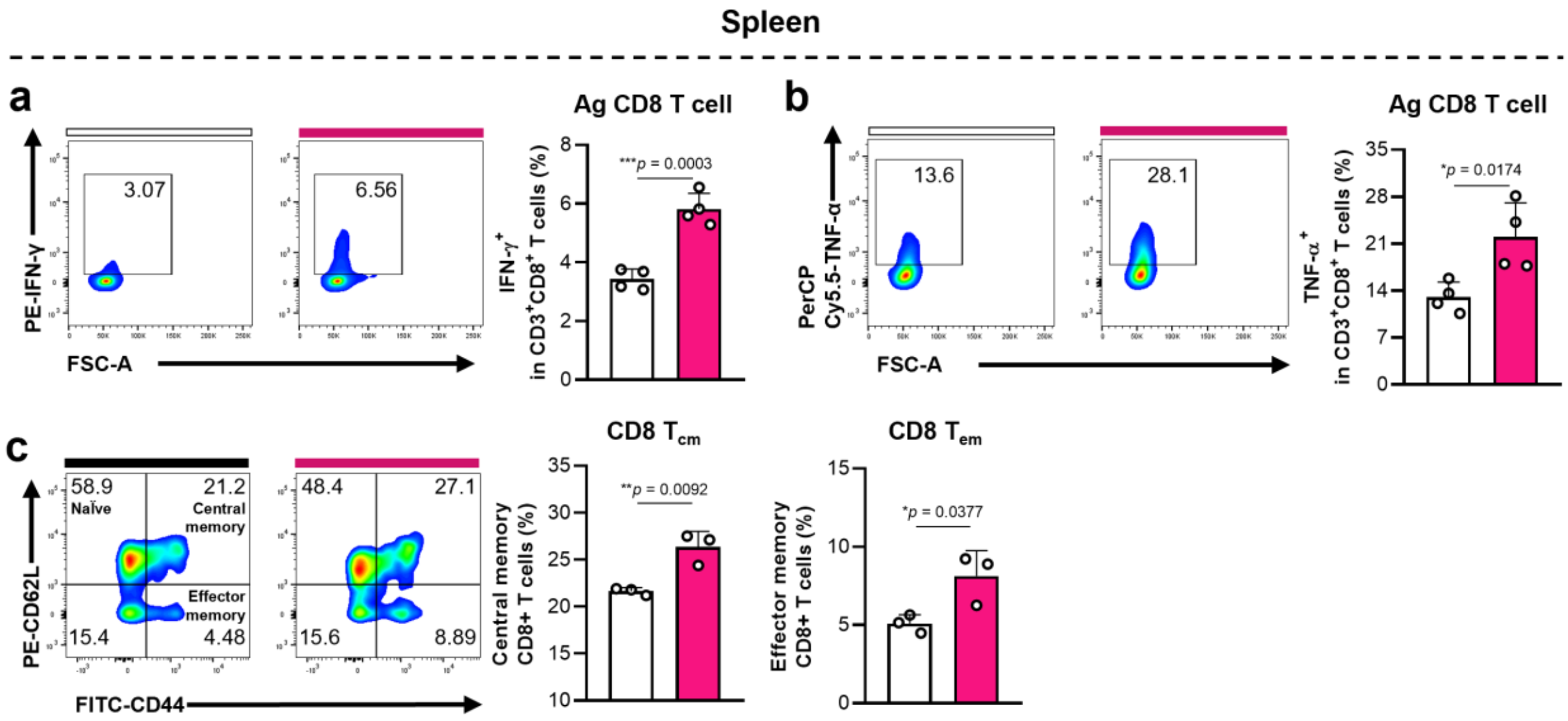

Figure S14. Splenic antigen specific CD8$^+$ T cell and memory CD8$^+$ T cell analysis from operated mice. a,b) Analysis of antigen-specific CD8$^+$ T cells secreting (a) IFN-γ and (b) TNF-α. c) Evaluation of central memory (CD8+ T_{cm}, left) and effector memory (CD8+ T_{em}, right) T cells in the surgical model (n=3 per group). All data are presented as the mean ± s.d. Statistical significance was evaluated by unpaired two-tailed t-test in a-c. *p*-values: $*p < 0.05$; $**p < 0.01$; $***p < 0.001$.

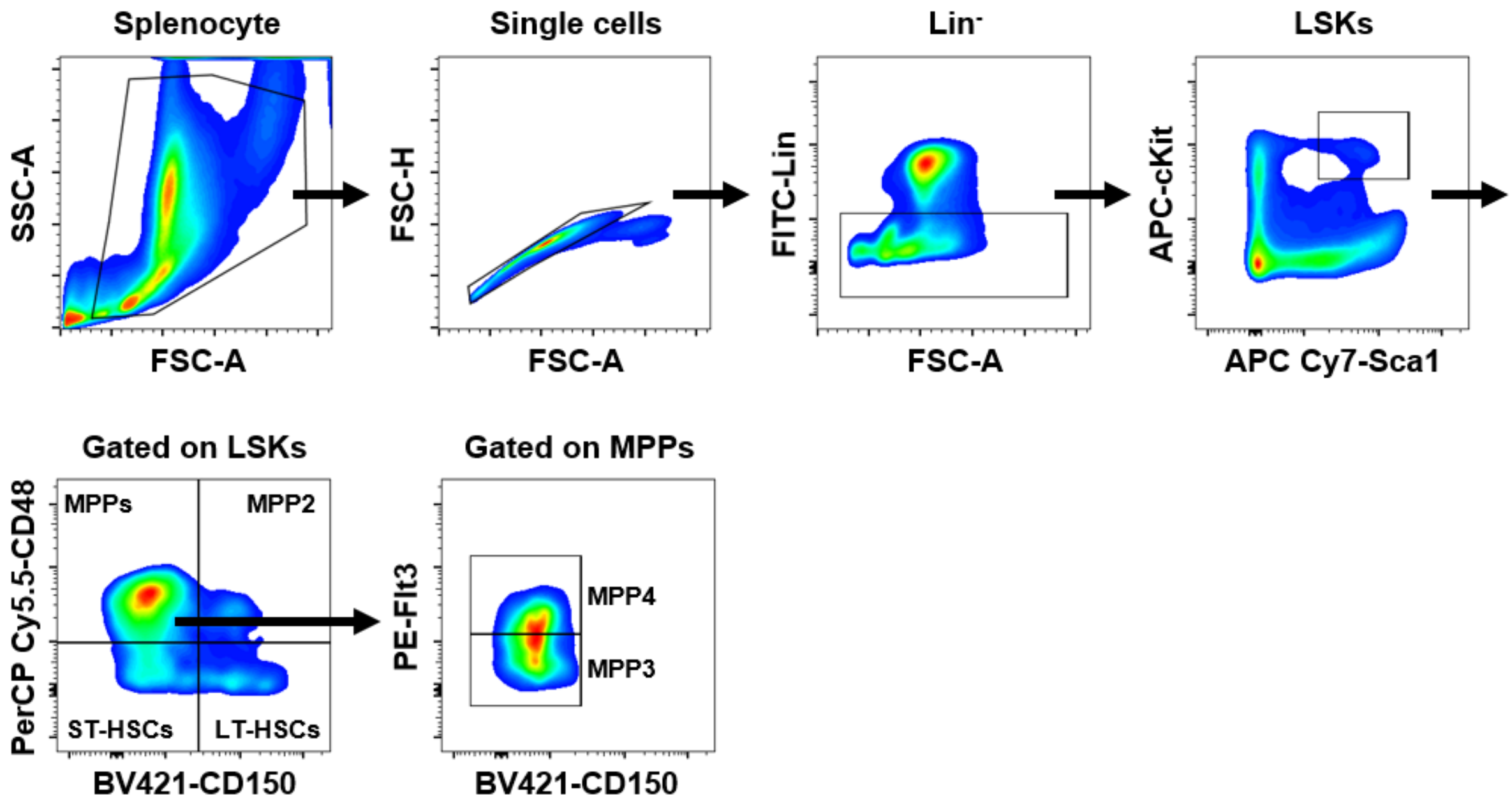

Figure S15. Gating strategies for analysis splenic HSPCs. Gating strategy for investigating the populations of splenic HSPCs.

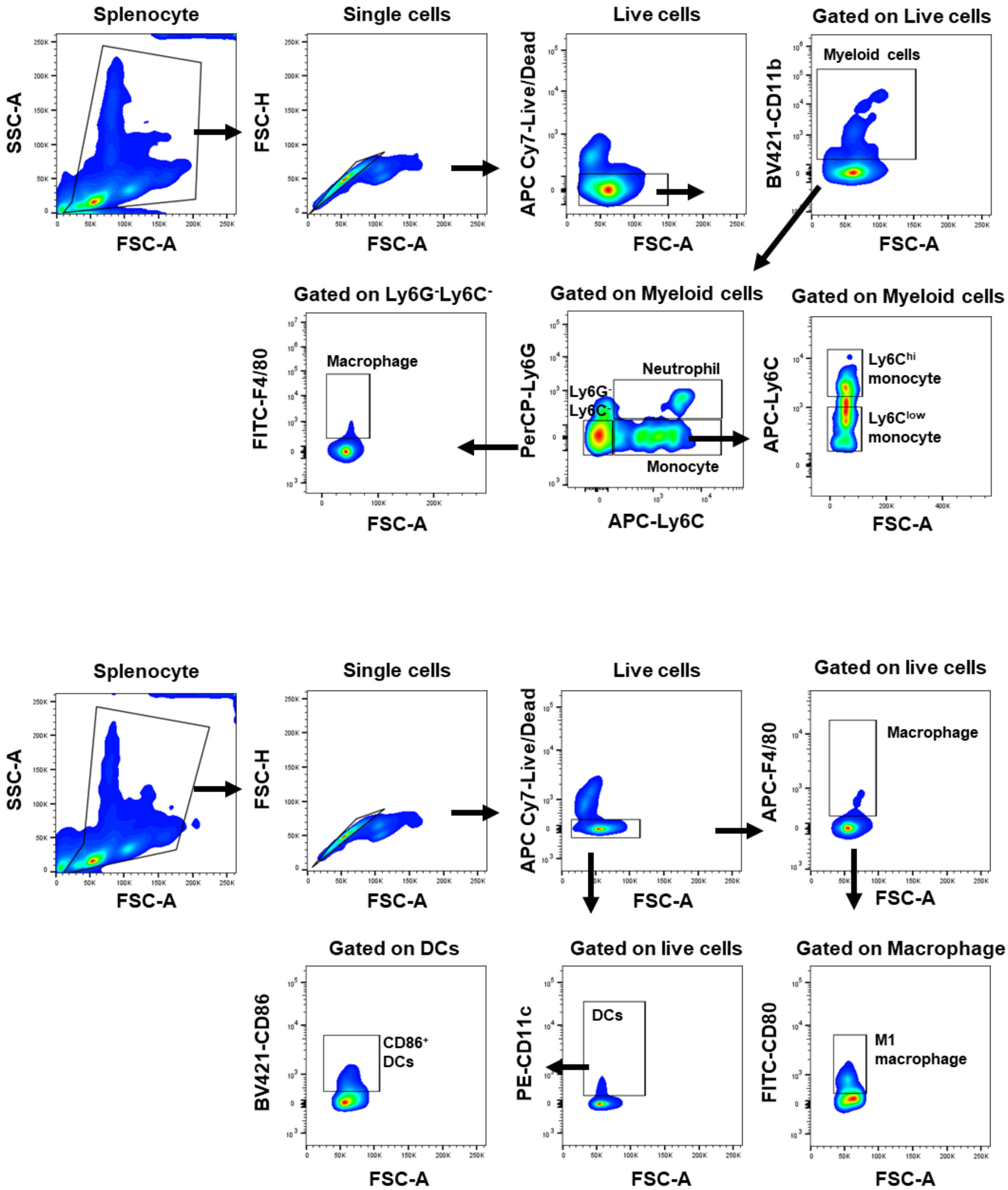

Figure S16. Gating strategies for analysis splenic myeloid cell population. Gating strategy for investigating the populations of monocyte, neutrophil, macrophage, and dendritic cells.

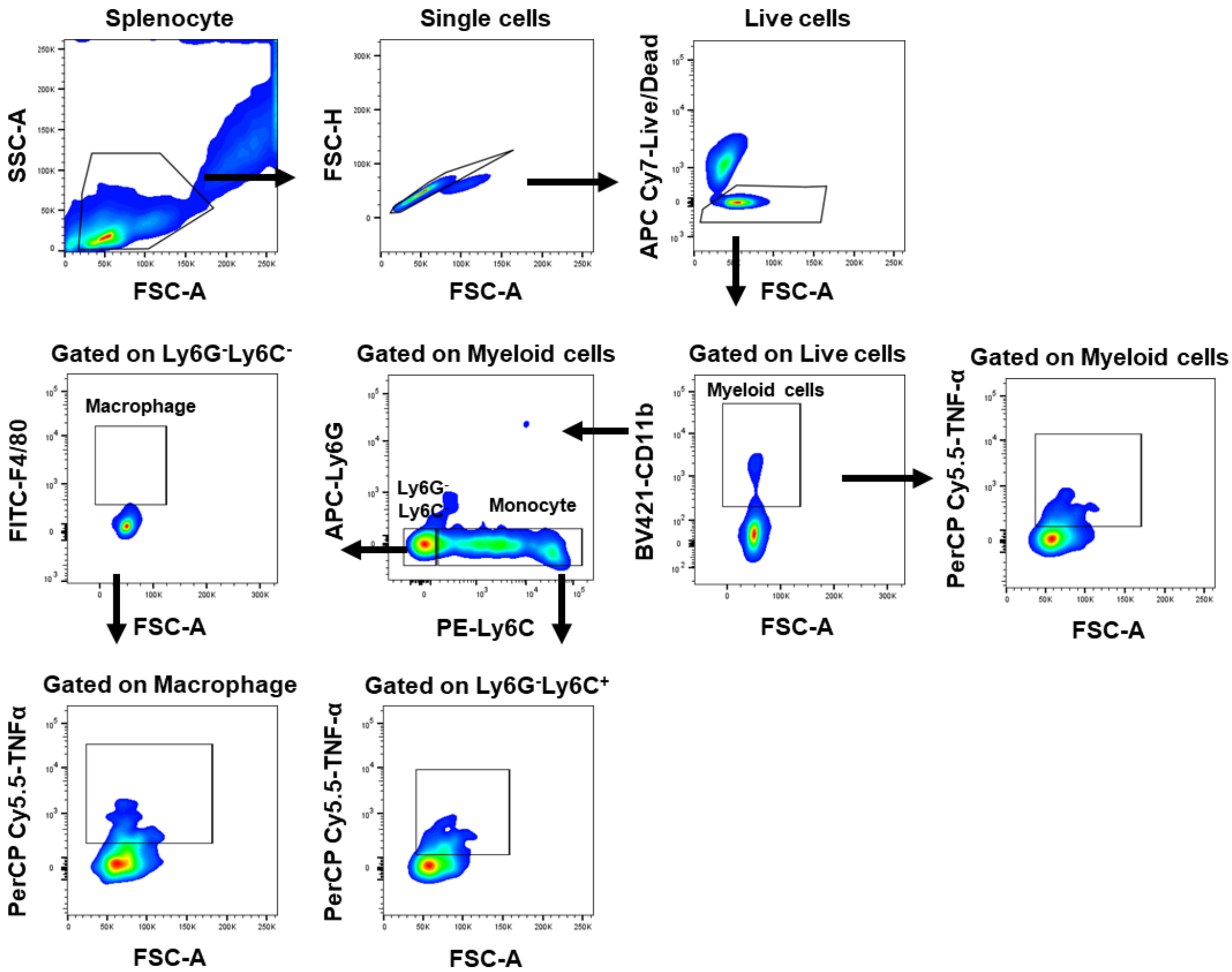

Figure S17. Gating strategies for analysis TNF-α+ splenic myeloid cell population. Gating strategy for investigating the populations of TNF-α+ myeloid, monocyte, macrophage cells.

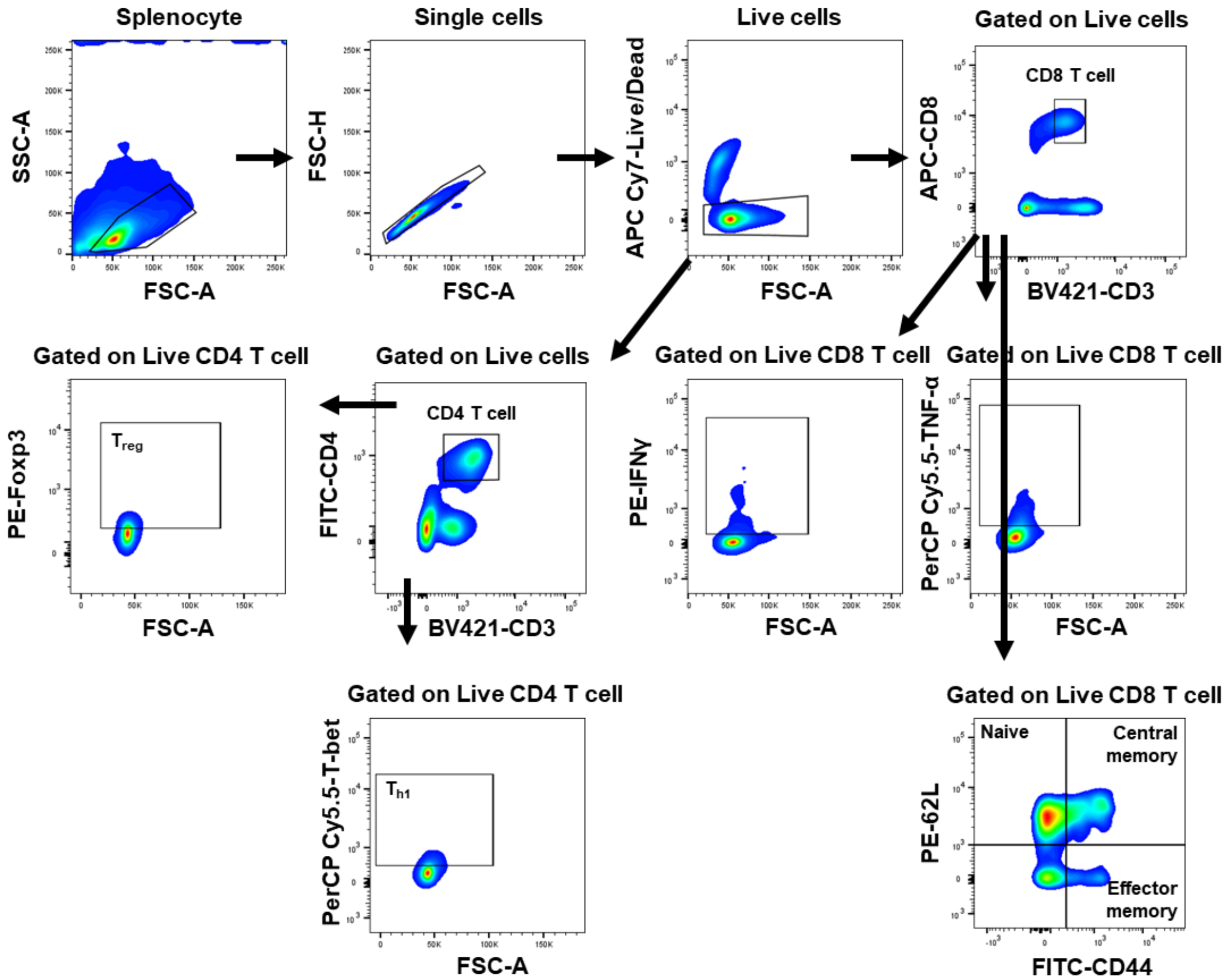

Figure S18. Gating strategies for analysis splenic adaptive immune cell population. Gating strategy for investigating the populations of T_{reg}, T_{h1}, antigen specific CD8 T cells, and memory CD8 T cells.

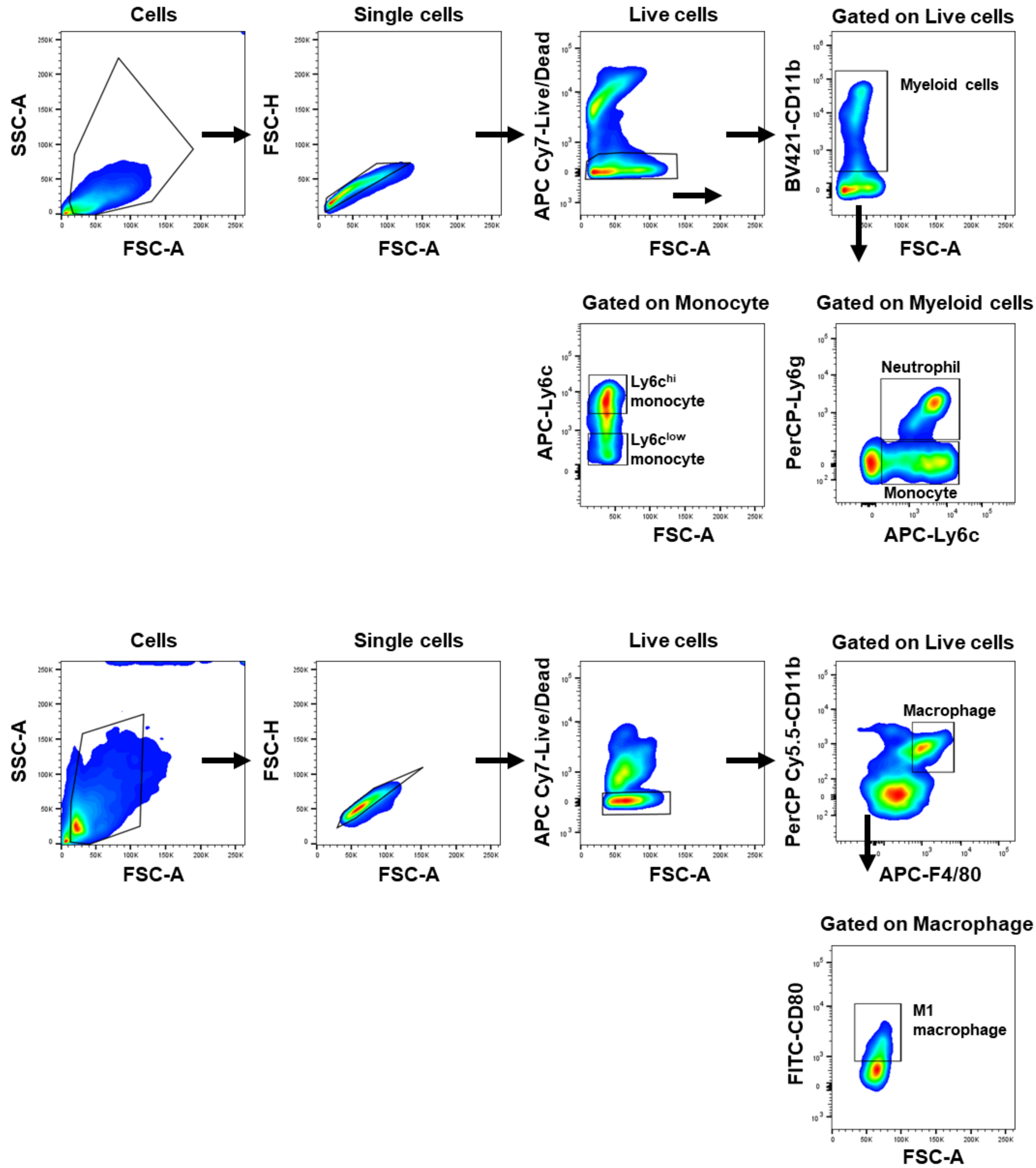

Figure S19. Gating strategies for innate immune cell population in TME. Gating strategy for investigating the populations of monocyte, neutrophil, and macrophage cells in TME.

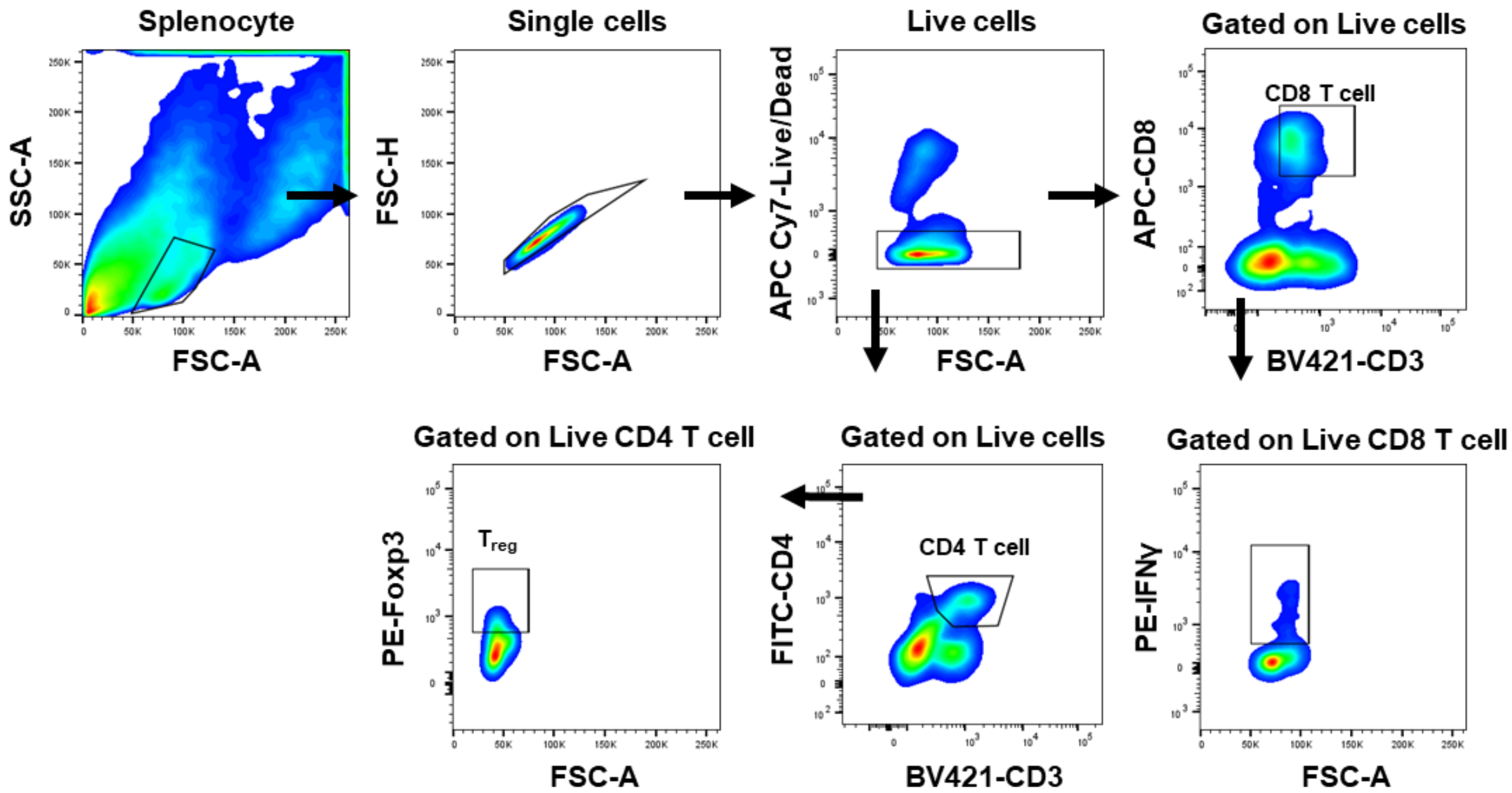

Figure S20. Gating strategies for adaptive immune cell population in TME. Gating strategy for investigating the populations of Treg and antigen specific CD8 T cells in TME.

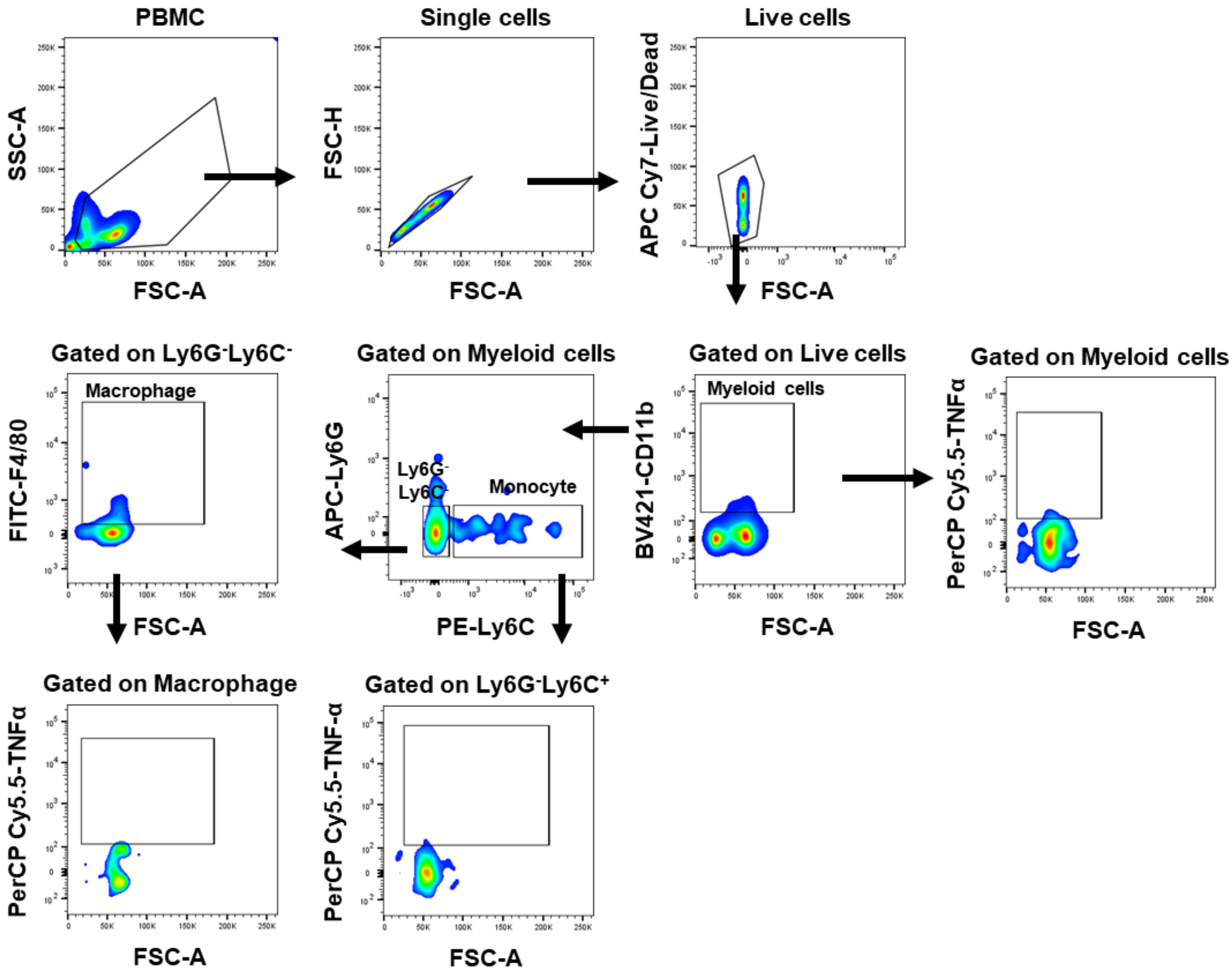

Figure S21. Gating strategies for analysis TNF-α$^+$ myeloid cell population in PBMCs. Gating strategy for investigating the populations of TNF-α$^+$ myeloid, monocyte, macrophage cells in PBMCs.

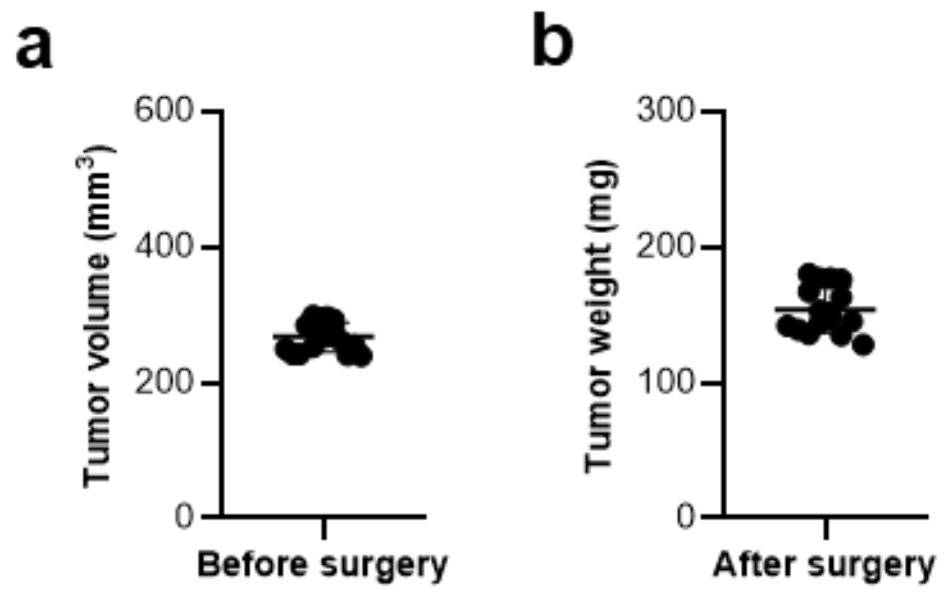

Figure S22. Tumor resection procedure. Strategy to control tumor resection size. a) Tumors were measured and randomly allocated for each group. b) Tumors were removed (approximately 90% of the primary tumor). Tumor removal was verified by measuring the residual tumor weight.
